# Self‐Powered Neuromorphic Touch Sensors Based on Triboelectric Devices: Current Approaches and Open Challenges

**DOI:** 10.1002/smll.202511256

**Published:** 2026-01-31

**Authors:** Fabrizio Torricelli, Giuseppina Pace

**Affiliations:** ^1^ Department of Information Engineering University of Brescia Brescia Italy; ^2^ Institute for Microelectronics and Microsystems National Research Council (IMM‐CNR) Agrate Italy

**Keywords:** neuromorphic sensor, self‐powered sensors, tactile sensor, triboelectric, tribotransistor, tribotronic

## Abstract

Advanced neuromorphic systems mimicking the human sensory and nervous system will enable artificial perception for intelligent robotics and human machine interfaces. Among sensing modalities, tactile perception is crucial for replicating human somatosensory and motor functions, with significant potential to restore impaired tactile capabilities. Artificial neuromorphic sensors can directly sense, store and process various stimuli information and implement computation functions such as perception, learning, and memory. However, computational energy efficiency must be achieved with novel neuromorphic systems capable of environmental energy harvesting enabling self‐powered sensing, and real‐time edge data processing. Here, we focus on the integration of tactile self‐powered sensors based on triboelectric nanogenerators (TENGs) with neuromorphic devices. We systematically discuss current approaches for coupling TENGs with artificial synapses and neurons, covering the main integration architectures (ex situ, discrete circuit, direct gating, monolithic), the primary operational modes (displacement‐driven, pulse‐driven), and neuromorphic functions as short‐ and long‐term plasticity, memory, and logic‐in‐memory computing. We also highlight the mechanisms of signal generation and transduction, and the strategies used to enhance performance and energy efficiency. The review concludes with a discussion on key challenges and future directions for developing sustainable, low‐power, and multifunctional neuromorphic tactile systems, paving the way toward fully integrated self‐powered artificial somatosensory platforms.

## Graphical abstract

This review outlines how triboelectric self‐powered tactile sensors can be integrated with neuromorphic devices to emulate human touch. It summarizes current coupling strategies, operational modes, and synaptic functions enabled by triboelectric nanogerator (TENG)‐based systems, while highlighting key mechanisms, performance considerations, and future challenges toward intelligent, low‐power artificial somatosensory platforms.

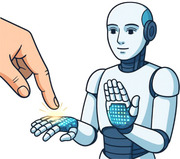

## 1 Introduction

Neuromorphic sensors based on standard electronics architectures present several limitations due to the physical separation between different system elements comprising the physical or chemical sensing unit, and the data storage and computing units [1]. The required signal processing for data transfer between these units carries the drawback of high energy consumption and encoding delays. An additional issue is represented by the high energy consumption required by computation at central nodes, that receive all the sensor raw data, which includes a large amount of redundant information. Such data should be pre‐processed at the sensor level to relieve the huge energetic consumption of the sensory computing system. However, current von Neumann architectures cannot ensure the high yield in processing and computing that should be performed at the sensor location [1], particularly when it comes to the need to collect different data inputs originated by edge sensors such as multi‐pixels or multi‐taxels arrays, as in vision and tactile sensing. Edge‐neuromorphic sensing and computing are emerging technologies aiming to relieve the burden of the large energy consumption and “data computation delays” associated to data processing and storage at central nodes, by delocalizing sensing and computation at the edge, i.e., where the analog data input (i.e., the sensor signal) is originated. Shifting neuromorphic architectures at the edge can ensure efficient large data set coding/encoding/transmission and filtering prior the computation at central nodes. Soft matter electronic engineering also promises to develop artificial neurons capable of integrating into flexible and conformable substrates, suitable for integration into edge electronics for soft robotics and prostheses [2, 3, 4].

Among different sensing functions, tactile sensing can cover a large variety of application fields, going from automotive to industrial manufacturing, and from robotics to prosthesis, holding a large potential impact on future technologies [5]. The biological tactile perception system is composed of numerous cells called mechanoreceptors each specialized in perceiving different types of mechanical inputs, such as sharpness, vibration and roughness, and transduce them into ionic electrical signals called action potentials. The human somatosensory system has evolved over time to perform edge neuromorphic sensing, which is at the base of the high efficiency of following brain computing [6]. Each biological mechanoreceptor is connected to afferent neurons, which pre‐process the sensing information before its transmission through the nerve fibers and synaptic connections, to the somatosensory cortex in the brain where the information is decoded. In another region of the brain, the motor cortex receives and sends feedback information, in the form of action potential, to the organs and muscles.

Artificial tactile sensors developed so far have exploited different transduction mechanisms and include capacitive, piezoresistive, piezoelectric and triboelectric sensors [7, 8, 9] Their integration with neuromorphic devices such as artificial synapses and neurons has already been shown to enable biomimicry of biological sensing and brain‐like logic functions. Tactile neuromorphic edge sensors will further reduce the energy consumption of artificial systems by enabling on‐site computation, and by preprocessing the mechanical inputs before their transmission to the next computational level, whether in the brain as in prosthetics or at the centralized computing hardware. This edge‐data preprocessing and filtering can realize the desired balance between high sensitivity and inadvertent triggering rate reducing computational burden. Integration of the artificial mechanoreceptor with neural networks for advanced information processing, object recognition, and actuation feedback were already presented in previous reviews, showing that these integrated systems not only can emulate human skin sensing but also the afferent/efferent system [10, 11, 12].

Though neuromorphic edge technologies hold the promise to establish groundbreaking innovation in future electronics, their effectiveness in supporting a sustainable transition to the new digital era might be limited by their requirement for power supply. Making edge sensors self‐powered is a necessary step for future electronics development, reducing the need for batteries and their overall carbon footprint [13]. The convergence of advances in low power electronics and innovative energy harvesters will enable the design and fabrication of edge sensors and devices that potentially could be fully self‐powered.

Among self‐powered tactile sensors, those based on triboelectric nanogenerators (TENGs, here also called TE‐sensors) [14, 15] have become a relevant player for harvesting low frequency, low forces and high‐entropy (stochastic) mechanical energy as typically originated from human, wave [16]. and wind motion [17]. TENGs have already been shown to be highly integrable in different cutting‐edge‐technologies such as in biomimetic artificial e‐skin, self‐powered touch and display panels for human‐machine interfaces, prosthetics, functional robotics and in biomedical devices [18, 19]. TENGs mechanical‐to‐electrical energy conversion mechanism relies on the natural triboelectrification phenomena occurring upon friction between two materials [20]. The triboelectric charge density harvested on the materials’ surface is then responsible for the establishment of an electrostatic induction, that enables the conversion into electrical energy of the mechanical input (section 3). A relevant property of TE‐sensors is the self‐powered and event triggered voltage output (tribopotential) originated by contact(touch) or release(detach) actions. TE‐sensors provide a self‐powered transduction of external mechanical stimuli enabling fast response, high flexibility and excellent stability. Moreover, their electrical output encodes rich information about the applied pressure, the frequency of stimulation and even the nature of the contacting materials.

The event‐driven response of TE‐sensors is well‐suited for interfacing with neuromorphic computing based on machine learning (ML) or artificial and spiking neural network (ANN and SNN) algorithms [21]. These software‐based frameworks emulate the human brain logic and computing by processing the digitized sensor analog output similarly to the action potential of biological neurons. Interfacing arrays of self‐powered TE‐sensors with ANN has shown promise for emulating brain‐like information processing, by enabling the interpretation and classification of complex analog signals acquired simultaneously from multiple sensing channels [22]. ANN interfacing with tactile neuromorphic TE‐sensors has already shown efficient data processing providing self‐learning, pattern, texture and material recognition with a high level of accuracy (>95%).

Despite the much higher processing speed of neural networks compared to conventional logic, a key step toward sustainable digital technologies lies in the development of neuromorphic sensing hardware that operates with ultra‐low power, enabling brain‐like computation with minimal energy consumption [1]. When a neuromorphic device, such as a memristors and/or a neuromorphic transistor, is coupled to a TE‐sensor, the tribopotential pulse can be quickly and efficiently transduced into a brain‐like pulsed neuromorphic signal output, realizing sensing and neuromorphic hardware integration. The emerging field of tribotronics explores the use of triboelectric potential to control the gate voltage in transistors, enabling the realization of tribotransistors [23, 24, 25]. This integration allows the mechanical event to be converted into a source‐drain current through the tribopotential modulation of the semiconductor channel conductance.

This review aims to present the various approaches that have been followed so far to integrate the TE‐sensors with the neuromorphic devices. Depending on the specific neuromorphic unit integrated with the TE sensor, distinct functional behaviors can be realized: (i) memory capabilities through devices such as memristors, biristors, charge‐trapping or ferroelectric transistors; (ii) synaptic plasticity via integration with ion‐gated transistors; and (iii) neuronal‐level signal processing when interfaced with multiple neuromorphic elements or dedicated neuron chips.

A primary classification of TE‐sensor/neuromorphic device integration can be made based on the system architecture, including: (i) hybrid configurations using discrete circuit components (such as diode rectifiers or oscillators) based on conventional silicon electronics, which serve as intermediaries between the TE‐sensor and the neuromorphic unit; (ii) direct ex situ connections, where the TE‐sensor is wired directly to the terminals of the neuromorphic device; and (iii) monolithic integration, in which the triboelectric sensing material is structurally and functionally incorporated into the neuromorphic device as an active element in a monolithic system.

Based on the mechanical input modality used to trigger the tribopotential signal delivered to the neuromorphic unit, two main operation modes can be distinguished: (i) displacement‐driven neuromorphic devices, where the relative distance between the triboelectric materials is incrementally varied to generate quasi‐static tribopotential steps, enabling controlled signal transmission to the neuromorphic unit; and (ii) event‐ (or pulse‐) driven devices, in which each individual mechanical interaction, such as a touch (press) or release (detach) event, produces a transient tribopotential pulse. The displacement‐driven mode has been predominantly employed for mechano‐programmed logic‐in‐memory operations and multibit neuromorphic computation [26]. The second modality is more appropriate for converting event‐driven tribopotential pulses into post‐synaptic current spikes, with temporal and amplitude features that closely resemble real tactile sensations.

State‐of‐art works demonstrate that it is possible to achieve real time human‐machine interactions, energy efficient data storage, and neuromorphic computing triggered by a self‐powered tribopotential. The integration of sensing, processing, and memory functionalities at the edge can greatly improve system efficiency by minimizing data transmission requirements, reducing the hardware footprint at central nodes, enhancing response speed through local computation, and lowering energy losses from long‐range data transfer. Multichannel signal transmission from multi‐sensor arrays has also been demonstrated, representing a key enabling technology for future applications in human–machine interfaces. Looking ahead, the convergence of multisensory neuromorphic data with low‐power neural network hardware and software will be essential to achieve truly energy‐efficient, brain‐like computation highlighting the need for seamless integration between self‐powered edge neuromorphic sensors and ultra‐low‐power neural processors.

## 2 Biological Versus Artificial Somatosensory System

### 2.1 Biological Mechanoreceptors and Tactile Encoding

Human glabrous skin hosts four main types of low‐threshold mechanoreceptors, based on highly specialized cells and corpuscles, each specialized in encoding specific aspects of tactile stimuli [6]. Merkel cell–neurite complexes (SA‐I) are slowly adapting (SA) receptors with small receptive fields, highly sensitive to static pressure and edges, and are primarily responsible for encoding fine spatial details such as texture and shape. Ruffini endings (SA‐II) are slowly adapting receptors with larger receptive fields, responding to skin stretch and contributing to the perception of hand posture and object manipulation force.

Meissner corpuscles (FA‐I) and Pacinian corpuscles (FA‐II) are fast‐adapting (FA) mechanoreceptors. Meissner corpuscles respond to low‐frequency vibrations and dynamic skin deformations, playing a key role in motion detection and slip, whereas Pacinian corpuscles are extremely sensitive to high‐frequency vibrations and encode very small dynamic perturbations over a wide area of skin. Together, these mechanoreceptors span a broad range of force amplitudes and temporal frequencies and exhibit characteristic adaptation times that translate static and dynamic touch into spike trains along the afferent nerve fibers.

In addition to low‐threshold mechanoreceptors, nociceptors and thermoreceptors provide complementary information on noxious mechanical stimuli and temperature, contributing to protective reflexes and multimodal perception.

These encoding strategies impose clear requirements for bio‐mimetic tactile systems: (i) high sensitivity and low detection thresholds to resolve gentle touch; (ii) a broad dynamic range covering both static and dynamic stimuli; (iii) tunable adaptation times mimicking SA and FA responses; (iv) spatial resolution comparable to mechanoreceptor receptive fields; and (v) the capability to convert mechanical inputs into event‐driven electrical signals that can be directly processed by neuromorphic circuits.

### 2.2 Afferent Neurons, Synapses and Neural Coding

**FIGURE 1:**
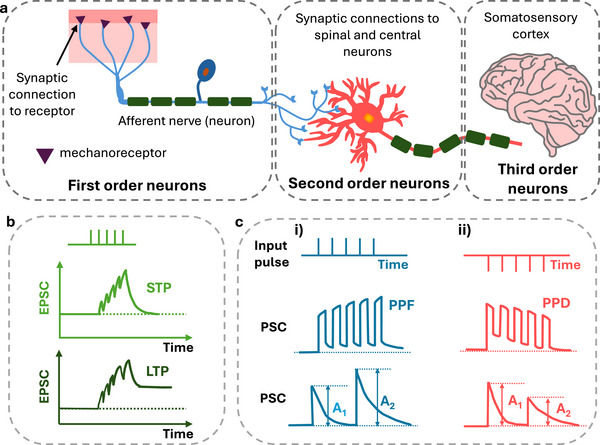
(a) Simplified scheme of the biological tactile perception system. (d) Scheme of the different possible features expressed by an STP response [33]. (b) Short term (STP) and long term (LTP) plasticity. STP shows a full recovery of the baseline current following the pulse excitation. In LTP the excitatory post‐synaptic current (EPCS) does not fully decay to the initial baseline current after pulse stimuli termination. (c) Post‐synaptic current (PSC) showing i‐ pair pulse facilitation (PPF) type of plasticity following a train of input pulses; ii‐ PSC showing pair pulse depression (PPD) type of plasticity following a pulsed input. The PSC increases during PPF strengthening the synaptic weights, while PPD inhibits the synaptic weights.

Once mechanical stimuli are encoded at the level of SA and FA mechanoreceptors, the resulting spike trains are transmitted through afferent neurons and synaptic connections to higher processing centers. The biological mechanoreceptor transduces the mechanical input into an ionic potential difference across the synaptic membrane, called action potential. The action potential constitutes the biological electrical signal transmitted across synapses and neurons and is at the base of all neural information processing and transmission. The sensory input stimulus generated by the mechanoreceptor (pre‐synaptic signal) is initially pre‐processed and computed at the level of the afferent nerve (sensory neuron) directly connected to the receptor (Figure 1a) [27]. The first layer of synapses and afferent nerves present on the skin decode and pre‐process the information received from the mechanoreceptor and delivers it in the form of post‐synaptic signal to the next neural connection. By doing so, the human body sensory system can rely on an extensive “edge‐computation” which is at the base of its high efficiency [28, 29]. The information that originates from such peripheral tactile signals’ integration and computation, is then transmitted through synaptic connections to the spinal neurons first, and then to the brain dense neurons network of the somatosensory cortex (Figure 1a). Here, the somatosensory neurons further compute and store the information received, and provide feedback to the motor cortex, whose function is to activate the body response, sending feedback signal to the muscles or organs, through the efferent nerve (motor sensor/actuator) [30]. In biological systems synapses receive inputs to one terminal (input neuron membrane) and deliver the output signal to another terminal (one‐in/one‐out). The memory (or encoding) function is more typically accomplished at the neuron's level, each connected to more than one synapse and neuron i.e., the neuron receives multiple signal inputs (many terminals for inputs), decode/encode the data and deliver/transmit multiple signals (many terminals for signal outputs, many‐in/many‐out) [31].

Learning and training in biological systems is mediated by the synapses that control the level (or strength) of information that is transferred between biological neurons. Synapse can be considered as a first level of information processing where the data input is decoded (elaborated, filtered, transduced) prior to reaching the next computational node. Synaptic plasticity refers to the synapses capability of strengthening or weakening the connection between neurons over time, in response to an increase or decrease of the input amplitude, dynamic and frequency [31]. The synaptic weight determines how the firing of action potentials from a presynaptic neuron influence the following neuron (postsynaptic neuron), and is defined as the strength of their connection or the strength of the information transmitted. Synaptic plasticity, where synaptic weights change based on a specific activity pattern, is essential to emulate the cognitive functions and adaptive behaviors typical of human learning, memory, forgetting and perception‐information processing, in neuromorphic computation and artificial intelligence. Computation at the single biological synapse level can already operate a variety of input signal processing functions, which primarily depends on the input pulse dynamics, and gives rise to short term (STP) and long‐term (LTP) plasticity (Figure 1b).

STP is responsible for perceptual and cognitive functions [32]. The STP behavior is expressed in the two typical behaviors of paired‐pulse facilitation (PPF) and paired‐pulse depression (PPD) (Figure 1c–e). Given a train of identical pre‐synaptic pulses, a PPF manifests with an increase in post‐synaptic current (PSC), that is observed when the synapse receives two subsequent pulses compared to a single pulse. In the presence of a PPD, the second stimulating pulse suppresses postsynaptic response compared to the first one. A PPF (or a PPD) index is therefore defined as the ratio between the amplitudes of the first (*A_1_*) and second (*A_2_*) PSC pulses (*PPF = A_1_/A_2_*). Most emerging devices exhibit positive feedback under stimulation i.e., PPF while the depression emulation is still limited [33]. PPF and PPD help to quantitatively evaluate the STP and are critical parameters for information processing function in neural network. The LTP causes changes in synaptic weight that last for several hours or longer, making it more strictly correlated with learning‐forgetting‐relearning functions of neuromorphic devices where data storage (memory) is required. LTP is evoked by continuous or multiple stimuli and is characterized by persistent facilitatory or inhibitory effects, explicated in long‐term potentiation or depression behaviors [34]. LTP and LTD can be expressed by spike‐timing‐dependent plasticity (STDP) [35], where the timing of action potentials (spikes) influences the strength of their synaptic connections. In many types of synapses, repeated presynaptic spikes that arrive a few milliseconds before postsynaptic action potentials typically result in long‐term potentiation (LTP), whereas spikes that arrive shortly after postsynaptic firing tend to induce long‐term depression (LTD) of the same synapses. This means that the relative timing of spikes determines whether the synapse strengthens or weakens. Another mechanism for inducing LTP or an LTD involves delivering action potentials to the synapse at varying rates or frequencies, giving rise to spiking rate‐dependent plasticity (SRDP) and spike frequency‐dependent plasticity (SFDP), respectively.

### 2.3 Artificial Neuromorphic Tactile Systems for Edge Computing

While synaptic interfaces are an essential feature to be emulated by artificial systems, they represent only a first step toward the far more complex task of reproducing the full local processing performed by the integrated system of biological mechanoreceptors and afferent neurons. Although still under investigation, current evidence shows that each specialized first‐order afferent neuron in the skin (fast adaptive, FA‐I or slow adaptive, SA‐I neurons) densely innervate multiple mechanoreceptors, enabling significant preprocessing before signals reach the somatosensory cortex. As a matter of example, it is known that the multiple connections to different receptors allow a single afferent neuron to deliver to the central cortex an informative signal that already contains edge orientation information [36, 37]. Optimal neuromorphic tactile systems shall emulate the entirety of the complex functions and preprocessing function of the first layer of the afferent neurons that densely innervate the glabrous skin. Therefore, while an artificial mechanoreceptor can perform mechanical signal transduction, truly human‐like neuromorphic sensing must account for the local integrative computational functions jointly carried out by mechanoreceptors and their associated afferent neurons.

**FIGURE 2:**
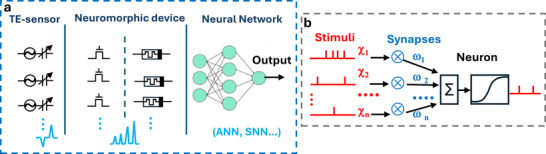
(a) Simplified equivalent circuits elements comprising artificial mechanoreceptors (TE‐sensors) and neuromorphic devices (synaptic transistors and memristors) emulating the first and second layer of the human peripheral somatosensory system and the brain neural cortex system emulated by neural networks (e.g. ANN and SNN). (b) Scheme of a spiking neural network (SNN) receiving different neural input (χ_i_) which are given specific synaptic weights through the synaptic coefficients (ω_i_) during the neural network training and learning process.

Overall, a comprehensive artificial neuromorphic system should be designed to emulate neuronal activity across multiple levels: at the device level, at the neural network level, and at the system level, thus encompassing interactive sensing and response to external environmental stimuli. Different neuromorphic device architectures have been designed to biomimicry the human somatosensory system and brain computation. To complete the process of bio‐mimic the human somatosensory system, artificial neuromorphic devices can be designed to accomplish one (as with single synaptic device) or more (with ensemble of artificial synapse and/or neurons) of the human neuro‐sensing functions. The afferent neurons are typically emulated by the direct connection of the artificial receptor (piezoelectric, piezoresistive, capacitive, triboelectric sensor) to a synaptic device which is connected to artificial neurons (Figure 2a). The neuromorphic processed information is then transferred to neural networks (as ANN and SNN) for further computation.

In neural networks, synaptic plasticity reflects how past experiences, encoded in synaptic coefficients (ω_i_), shape the neural input (χ_i_) (Figure 2b). Therefore, synaptic weights modulate the amplitude of the signal transmitted across the synapse, playing a key role in learning and memory functions. The synaptic plasticity of artificial devices is based on the modulation of the device conductance or capacitance and, in analogy to the biological counterpart, it is distinguished as STP and LTP [38]. The STP is activated by transient stimuli that causes rapid changes in synaptic weight (typically from millisecond to seconds) allowing the synapse to quickly recover the unstimulated state.

The scope of artificial edge sensing is to integrate multiple neuromorphic functions and computations at the edge, reducing data latency and allowing real‐time processing of input signals. This approach results in overall energy savings and improvement in data computation efficiency. However, delivering the transduced signal from the artificial mechanoreceptors to the neuromorphic device is not as simple as just connecting them with wires. Each component must be carefully designed to ensure the highest performance and energy efficiency of the integrated system. A general scope in the development of neuromorphic devices is to improve the device performance by increasing switching ratio and speed, miniaturizing the components, and reducing power consumption by lowering operational voltage or current [39].

## 3 TE‐Sensors as Bionic Mechanoreceptor

Among the biological mechanoreceptors [27], Merkel cells have specialized for light touch sensation. Ruffini corpuscles are specialized to control finger position and movement performing static sensing. Pacinian corpuscles sense vibrations and deep pressure only when the skin is indented rapidly but are not sensitive when pressure is steady. Meissner corpuscles are cutaneous nerves that transmit fine discriminative touch and vibration (as skin indentation with frequency of 10–50 Hz), and they trigger the release of nociceptive effectors for pain sensation (spatial resolution 3–5 mm). A major challenge in the field of edge tactile sensing is the integration of the various functional roles of different biological mechanoreceptors into a single, or a few highly integrated, artificial tactile sensors. Artificial mechanoreceptors are still far from being capable of emulating all the above biological functions. The ones developed so far mostly mimic the biological sensation of either static or dynamic pressure, mostly emulating the Merkel and Pacinian cells, respectively. Artificial nociceptors [40], based on neuromorphic TE‐sensors have also been successfully demonstrated and are described in Section 8.2.

From the viewpoint of artificial mechanoreceptors, TE‐sensors naturally emulate fast‐adapting (FA) behavior, as their output is dominated by transient tribopotential peaks associated with contact‐and‐release events or dynamic deformation. This makes them highly suitable for encoding dynamic touch, vibration and slip, analogous to Meissner and Pacinian corpuscles. Nevertheless, future generations of self‐powered devices are expected to evolve beyond isolated FA or SA emulation [41]. Mimicking slowly adapting (SA) responses requires strategies to maintain a stable output under static loads. This can be partially achieved by hybridizing TE‐sensors with capacitive, resistive or potentiometric elements that provide static readout, or by exploiting potentiometric components monolithically integrated within the TE structure. However, these approaches often introduce continuous power consumption and complexity at the circuit level.

Additional challenges specific to triboelectric mechanoreceptors include the dependence of surface charge density on environmental conditions (e.g., humidity and temperature), the non‐linear relationship between applied force and tribopotential over broad force ranges, and the unipolar nature of the triboelectrode polarization, which can limit symmetric program/erase operations in neuromorphic devices. On the other hand, TE‐sensors offer unique merits: they are self‐powered and event‐driven, compatible with a wide range of flexible and biocompatible materials, scalable to large‐area arrays, and capable of encoding rich information on force amplitude, frequency and contact mode in a single output waveform. These features make TE‐sensors particularly attractive building blocks for neuromorphic mechanoreceptors, provided that their intrinsic limitations are addressed through materials, device and system‐level design.

Tactile receptors based on capacitive, piezoresistive or resistive elements are prone to emulate static pressure sensitivity, while the event‐based spiking response of TE‐sensors is more effective at emulating dynamic tactile sensing [12]. However, capacitive and resistive sensors require the generation of sequential and periodical electrical pulses that are necessary to probe and map the force spatiotemporal properties, thus dissipating non‐negligible amounts of power even in absence of external stimuli. On the other side, piezoelectric and triboelectric sensors can provide a self‐powered sensing, relieving the environmental burden associated with battery usage [13, 42, 43]. However, efficient piezoelectric devices are mostly based on non‐sustainable materials such as lead‐based ceramics, particularly those based on MEMs technology [44, 45]. Additionally, they can ensure optimal energy conversion efficiency if they are operated at their resonant frequency which is typically above 50 Hz, being not compliant with human tactile sensing [46]. Compared to other artificial mechanoreceptors, TE‐sensors necessitate sufficient active contact area which limits their spatial resolution, however they can efficiently convert mechanical energy also in the low force and low frequency ranges, and in presence of stochastic events, fiercely competing with other self‐power sensor technologies.

**FIGURE 3:**
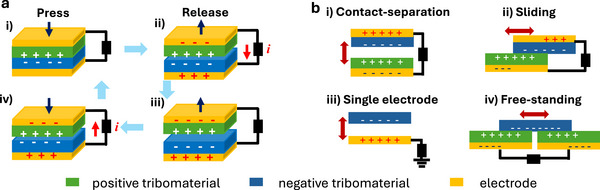
(a) Mechanical input transduction mechanism of two‐electrodes TENGs working in contact separation mode. (b) Scheme of the main operational modes and device configurations used for TENG: i‐ contact‐separation; ii‐ sliding mode; iii‐ single electrode; iv‐ free standing.

The most common TENGs are based on a two electrodes configuration operating in contact‐separation mode (Figure 3a). Here, two main structural components are present, i‐ a positive triboelectrode formed by a positive triboelectric material (tribomaterial) and its electrode and ii‐ a negative triboelectrode formed by a negative tribomaterial and its electrode. Upon cycling the TENG, and under contact conditions, a triboelectrification charging at each tribomaterial surface occurs, and an electrostatic field is generated (Figure 3a). When the two tribomaterials are separated, the variable electric field established in the airgap between the two triboelectrode triggers the flow of an induction current through the external circuit. An opposite current flow is recorded when the two triboelectrodes are reapproached. Per each mechanical cycle two current (or voltage) peaks opposite in sign are recorded corresponding either to the contact or to the release event. Due to the need to maximize the density of triboelectric charges accumulated upon contact, tribomaterials that reside at the opposite side of the triboelectric series are most suitable to be integrated into TE‐sensors [15, 47] A main advantage of TE‐sensors rely on their sustainable production [48], and the wide flexibility [49] of their device structure and geometry (Figure 3b) making it possible to harvest different mechanical motions (like sliding or rotatory). Compared to the two electrodes configuration (Figure 3b‐i), the single electrode one (Figure 2b‐iii) is the simplest structure for a TE‐sensor, allowing easier integration into electronic devices. However, it suffers from a lower sensitivity, thus most of the artificial neuromorphic mechano‐sensors reported so far are based on TENGs operating in the two‐electrodes configuration [50].

Recently, to more accurately mimic the biological somatosensory systems, TE‐sensors have been integrated into multimodal and hybrid sensing platforms. Acting as highly sensitive, real‐time, self‐powered active dynamic sensors, they are often combined with conventional static pressure sensors, such as flexible dual‐mode triboelectric‐capacitive‐coupled tactile sensor [51], and resistive sensors [52].

Strategies to increase the sensitivity of TE‐sensors have been largely reported in previous reviews [53, 54]. Typical two electrode TENGs integrate a metal electrode (as Cu or Al) acting as positive tribomaterial and a negative tribomaterials based on silicon rubber, e.g. Ecoflex and polydimethylsiloxane (PDMS), or fluorinated polymers, e.g., fluorinated ethylene propylene copolymer (FEP) or polytetrafluoroethylene (PTFE), polyvinylidene fluoride (PVDF) [55, 56, 57]. These polymeric materials are all residing at the negative side of the triboelectric series thus, they have a strong tendency to charge negatively. To boost contact electrification a common strategy is to increase the contact surface area by nano or micro structuring. [47] Practical challenges remain, in terms of material wear, temperature and humidity degradation, and stability over long‐term mechanical cycling. To address these issues surface treatments, structural design optimization, and encapsulation methods have been explored. Materials like PTFE and FEP are favored for their triboelectric properties and durability. Strategies to boost performance have included the use of nanomaterial fillers (e.g., graphene, 2D Materials, high dielectric constant oxides) [58, 59, 60, 61]. while self‐healing polymers enhance mechanical and chemical stability [54]. Ultimately, the synergy between design, material, and system integration determines the performance of TENG‐based artificial synapses for intelligent sensing and neuromorphic applications.

## 4 Neuromorphic Devices

**FIGURE 4:**
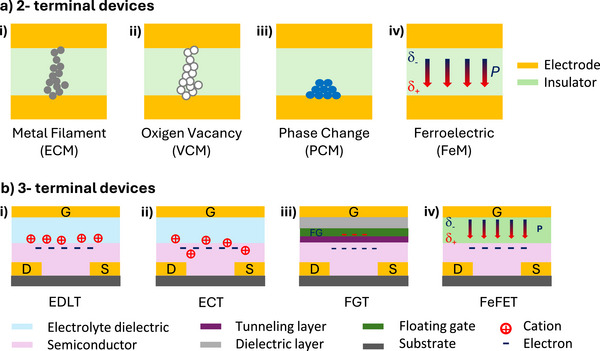
Schematic structure of the most common neuromorphic devices (gate, G; drain, D; source, S). (a) 2‐terminal devices: i) electrochemical memory (ECM); ii‐ valence change memory (VCM); iii‐ phase change memory (PCM); iv) ferroelectric memory (FeM). (b) 3‐terminal devices: i) electrical double layer transistor (EDLT); ii) electrochemical transistor (ECT); iii) floating gate transistor (FG‐FET); iv) ferroelectric transistor (FeFET). (3‐terminal schemes assume the presence of an *n*‐type semiconductor).

A distinction of neuromorphic devices is made between two terminals (memristors) and three terminals (neuromorphic transistors) devices, where the device conductance (or resistive) modulation and switch is used to process and/or store data and to emulate biological synapses and neurons (Figure 4) [62, 63]. The two architectures mainly differ on the input signal processing and device conductance modulation to control the level (or strength, viz. synaptic weight) of information sent through their output, mostly delivered in the form of PSC.

### 4.1 Two‐Terminal Neuromorphic Devices

Typical two terminals neuromorphic devices are memristors and capacitors. They are based on a metal‐insulator‐metal structure (MIM), where the input electrical signal causes a change in device resistance or capacitance. Memristors include resistive random‐access memories (RRAM), phase change memories (PCM), ferroelectric memories (FeM) and magnetic random‐access memories (not discussed here) [39]. In RRAM, the device conductance can be switched by electrical input stimuli which triggers a reversible chemical or physical process. This process leads to the formation or rupture of a conductive filament due to the migration of metal atoms in electrochemical memory (ECM) or oxygen in valence change memory (VCM). In PCM a material phase change is induced by joule effect, leading to a resistive switching of the devices, while in FeM, a reversible polarization of the ferroelectric material is controlled by the applied bias. Two‐terminal devices are mostly bi‐stable devices, characterized by a high resistance (HRS) and a low resistance (LRS) state, that can be set/reset by switching the voltage applied to the device. The high or low resistive states can be retained for a long time without the need for constant power supply, ensuring non‐volatile information storage, low power consumption, and high integration density. However, this feature limits the number of bits that can be accessed with the voltage writing, making memristor less suitable for multibit data storage.

### 4.2 Three‐Terminal Neuromorphic Devices

Due to their 3‐terminal nature, and the channel conductance modulation achieved through the control gate bias, neuromorphic transistors provide a larger flexibility in states tuning compared to memristors. However, compared to MIM structures, the multibit data storage achieved comes at the cost of a required continued power supply to maintain the information in the selected conductance state. Artificial synapses based on transistors (synaptic transistors) typically use the gate and the semiconductor as analogs of the presynaptic and postsynaptic membranes respectively. Different transistor terminals are used for signal transmission (drain‐source voltage, V_D_), conductance updating (gate‐source voltage, V_G_, presynaptic input) and signal output (drain source current, I_DS_, or postsynaptic current I_PSC_).

In synaptic tribotransistors, the I_PSC_ signal amplification is obtained by coupling the tribopotential with the semiconductor channel conductance. When the tribopotential pulse sent to the gate exceeds the transistor threshold voltage (Vth), charge carriers are injected at the source. Synaptic transistors (Figure 4b) so far integrated into a neuromorphic tribotransistor, are mostly based on electrolyte‐gated transistors (EGT), ferroelectric transistors (FeFET) and floating gate transistors (FGT). Among different field effect transistors (FETs), the EGT can operate at very low voltages (< 1 V) [64]. In EGT (Figure 4b‐i‐ii) the dielectric layer is based on an ionic layer where ions can drift under the influence of the gate bias. The channel conductance can be modulated by ionic dielectric such as ionic liquids, polyelectrolytes and proton conductors. The presence of ionic migration makes EGT more similar to biological synapses than other neuromorphic devices, closely mimicking the mechanism and dynamics of action potential. Different semiconductor materials have been so far investigated as 2D‐materials, organic materials and metal oxides. Depending on whether the ions interact with the semiconductor material [65], EGTs can be classified into two types: (i) electrical double layer transistors (EDLTs, Figure 4b‐i), which are electrostatically controlled by the formation of an electrical double layer (EDL) at the electrolyte/semiconductor interface, and (ii) electrochemical transistors (ECTs, Figure 4b‐ii), in which the ions penetrate into the semiconductor, leading to a bulk ionic‐electronic modulation. In EDLT, when a voltage bias is applied to the gate (*V_G_*), ion migration leads to the formation of an EDL with an ultrathin thickness (∼ 1 nm) at both the gate/dielectric and dielectric/semiconductor interfaces. Due to the high EDL capacitance (∼ 10 µF/cm^2^), EDLTs can operate at low voltages (few mV) thus ensuring low energy consumption (pJ‐fJ). For a n‐type semiconductor when a positive gate potential is applied, the anions redistribute along the dielectric layer moving toward the interface with the gate electrode, while cations drift in the opposite direction toward the semiconductor channel (Figure 4b‐ii). According to the reversibility of the ion induced conductance switch, different plasticity can be inferred to the synaptic transistor [62]. The EDL formation is responsible for the electron injection into the semiconducting channel and the detection of an excitatory post‐synaptic current (EPSC). Since ions are low‐mobility charges, the EDL formed under tribopotential pulse will be maintained even after the mechanical input is removed. This results in a time dependent decay of the EPSC, which is influenced by the ionic mobility. As a result, the basic synaptic function of STP can be emulated. The presence of long lived (more than seconds) ions residing in the semiconductor can allow for LTM functions.

Floating‐gate field synaptic transistors (FG‐FET or charge trapping FETs) are typically based on charge trapping neuromorphic transistors that incorporate an additional gating layer into a more conventional transistor architecture (Figure 4b‐iii). Typically, the floating gate is a charge trapping layer embedded between a dielectric insulator layer and a tunneling dielectric layer (with ultrathin thickness < 10 nm) that allows the charges in the semiconductor channel to tunnel toward the floating gate. The charge injection and storage into the floating gate is responsible for establishing an electric field that is opposite in sign to the gate field, causing a shielding effect. The charged trapping layer causes the shift of the threshold voltage (Vth) during the V_G_ scan, and a hysteresis appears in the transfer curves. The Vth shift is unidirectional (either positive or negative) depending on the majority carrier type in the channel. The slow (from milliseconds to seconds) recombination dynamics of the trapped charges, which flow back through the tunneling layer to recombine within the semiconductor channel, mimic the transmission process of the neurotransmitters of biological synapses. The de‐trapping process enables non‐volatile memory, which is crucial for emulating the LTP behavior of synapses, a key mechanism for learning and memory. The extensive development of FGT is limited by their high operating voltages (> 20 V) and long decay time (10–100 ms) limiting the fast writing/erasing and data storage.

In synaptic transistors based on van der Waals (vdW) heterostructures (vdW‐FETs), 2D layered materials are employed as semiconductors [66]. The combination of 2D‐materials with different bandgaps allows the tuning of the electrical and/or optical properties of the channel, enabling the simulation of multiple functions within a single artificial synapse. Neuromorphic transistors integrating vdW heterostructures have been shown to both boost channel conductivity and to improve charge trapping efficiency in FG‐FET allowing multilevel memory function.

In FeFETs (Figure 4b‐iv), a ferroelectric material, such as bulk perovskite, doped hafnium oxide (HfO_2_), or piezoelectric polymers, serve as the dielectric insulator [67]. These materials have the unique ability to exhibit a reversible polarization when subjected to an external electric field, allowing the modulation of the transistor channel conductance. When a bias voltage is applied to the gate, the ferroelectric material polarization is induced, creating an electric field that alters the conductivity of the channel and, enabling the device to act as a synapse [68]. sUpon removal of the gate voltage, the induced material polarization remains, a characteristic that allows FeFETs to store non‐volatile information, providing the system with a persistent memory that can be read at any time and making them suitable computational tasks. When a positive or negative gate bias is applied, the ferroelectric layer becomes polarized in one direction or the other, effectively “writing” or “erasing” the information [69]. The ability to reverse the polarization with an opposite bias further enables the erase functionality, making FeFETs well‐suited for simulating synaptic plasticity, such as LTP and LTD, both of which are critical for learning and memory in biological systems [70].

However, the presence of charge traps and gate leakage currents can reduce the state retention time, posing a challenge for the long‐term stability of the stored information. To address this issue, emerging ferroelectric semiconductors based on 2D‐layered ferroelectrics, where polarization occurs directly in the channel, are being explored. These materials offer the potential to improve polarization retention and reduce the impact of leakage currents and charge trapping.

While memristors are traditionally favored for neuromorphic memory applications due to their ability to retain information without the need for constant power, 3‐terminal devices based on FeFETs or FG‐FETs can be designed to accomplish both synaptic and memory functions. These devices can combine both synaptic behavior and memory retention within a single component, providing a more compact and efficient approach to building neuromorphic systems. The integration of these devices into neuromorphic architecture holds great promise for advancing brain‐inspired computing systems that can perform complex cognitive tasks while maintaining low power consumption.

Three‐terminal neuromorphic devices offer complementary trade‐offs when operated under triboelectric gating. EDLTs and other ion‐gated transistors exploit the formation of an electric double layer to achieve very high gate capacitance and strong channel modulation at low voltages. Their response is intrinsically linked to ion migration dynamics, which enables short‐term plasticity and gradual transition to long‐term plasticity, but also limits switching speed and long‐term stability due to ionic drift and possible electrochemical reactions in the dielectric or at the channel interface.

Electrochemical transistors (ECTs, including OECTs) further rely on volumetric doping of the channel. They exhibit large transconductance and strong analog weight modulation at low operating voltages, which is advantageous for realizing continuous synaptic weight updates driven by TE‐pulses. However, the volumetric ion penetration also makes them more sensitive to cycling‐induced degradation and to the composition and stability of the ionic medium.

Floating‐gate transistors (FGTs) and charge‐trapping FETs provide non‐volatile memory by storing charges in an embedded floating gate or trapping layer. When coupled to TE‐sensors, they enable long‐term plasticity and multilevel memory states, which is attractive for logic‐in‐memory and pattern recognition. Their main limitations are the typically high program/erase voltages, limited endurance, and the need for precisely controlled tunnelling dielectrics, which must be carefully matched to the amplitude and polarity of the tribopotential.

FeFETs leverage the remanent polarization of a ferroelectric layer to provide non‐volatile, fast and energy‐efficient switching. Under triboelectric gating, FeFETs can convert discrete mechanical events into persistent conductance states with low energy per operation, making them promising candidates for edge neuromorphic computing. Challenges lie in ensuring stable polarization switching under the irregular (ac‐like) tribopotential waveforms, mitigating charge trapping and leakage that degrade retention, and integrating ferroelectric materials with flexible and low‐temperature substrates.

Overall, EDLTs and ECTs are best suited for implementing tunable synaptic plasticity and sensory preprocessing at the TE‐sensor level, whereas FGTs and FeFETs are more appropriate for long‐term, multibit storage and logic‐in‐memory functions. Hybrid architectures that cascade or co‐locate these device types offer a promising route to fully exploit the rich temporal information encoded in triboelectric signals.

Table 1 compares commonly employed channel materials, triboelectric polymers, electrode choices, and functional gate layers across different three‐terminal neuromorphic architectures. Key advantages and limitations are highlighted to illustrate material‐dependent trade‐offs in sensitivity, stability, switching behavior, and compatibility with TE‐gating. The table also highlights key advantages and limitations of each material category, providing practical guidelines for device and system co‐design of TE‐regulated neuromorphic sensors.

**TABLE 1:** Summary of representative material systems used in triboelectric mechanoreceptors and three‐terminal neuromorphic devices.

Device Type	Channel / Active Material	Electrode / Gate Functional Layer	Advantages	Limitations and Challenges
TE Sensor (TENG‐based mechanoreceptor)	Tribomaterial: PTFE, FEP, PDMS, Ecoflex, cellulose‐based biopolymers	Electrode: Metal or carbon electrodes	‐ High surface charge density; ‐ Extreme device mechanical flexibility; ‐ Scalable fabrication; ‐ Self‐powered, event‐driven output ‐ high sensitivity for low force loads;	‐ Humidity‐induced surface charge screening; ‐ limited linearity response region; ‐ Material ageing or contamination; ‐ low sensitivity for high force loads;
EDLT (Electric Double Layer Transistor)	2D semiconductors (MoS_2_, WSe_2_), IGZO, conjugated polymers (DPP, P3HT)	Dielectric: Ion gels, polymer electrolytes, ionic liquids	‐ Very low‐voltage operation; ‐ Large gate capacitance; ‐ STP‐LTP transition due to ionic dynamics	‐ Slow ion diffusion (limited speed); ‐ Stability of electrolytes; ‐ Possible electrochemical reactions at channel /eletrolyte interface;
ECT / OECT (Electrochemical Transistor)	PEDOT:PSS, OMIECs, IGZO‐hybrids	Dielectric: Polymer electrolytes, hydrogels	‐ Mixed ionic‐electronic conduction; ‐ Large transconductance; ‐ Strong synaptic weight modulation;	‐ Material swelling, drift, cycling degradation;
FGT / Charge‐trap FET	Si, IGZO, MoS_2_, polymer semiconductors	Floating gate: charge‐trapping layer, high‐k oxides	‐ Non‐volatile storage; ‐ Multilevel memory; ‐ Compatibility with TE pulses for long‐term plasticity;	‐ High programming voltage; ‐ Reliability of tunnelling and blocking layers; ‐ Charge leakage; limited endurance;
FeFET (Ferroelectric FET)	Si, IGZO, 2D semiconductors	Ferroelectric: HfO_2_‐based layers, P(VDF‐TrFE)	‐ Low‐energy non‐volatile switching; ‐ Fast operation; ‐ Robust LTP; ‐ Suitable for edge computing;	‐ Polarization fatigue; ‐ Retention degradation under irregular tribopotentials; ‐ Integration on flexible substrates can be challenging.

## 5 Neuromorphic TE‐Sensor Integration and Operation Modes

The TE‐sensor unit mimics a biological mechanoreceptor which transduces the external mechanical input into a presynaptic electrical signal in the form of event‐driven tribopotential pulses. The basic neuromorphic artificial tactile sensing requires the integration of the TE‐sensor with a synaptic device capable of transducing the tribopotential (*V_TE_*) into a PSC. A relevant matter for optimizing novel neuromorphic devices is the energy dissipation associated with the generation of a post‐synaptic signal. The biological neuronal system is capable of large‐scale parallel signal processing at ultra‐low power consumption, and the energy dissipation of biological synapses is close to 10 fJ, a challenging target to be achieved with artificial synapses [71]. In synaptic transistors, the energy dissipation (*E*) of a single spike event is determined by the peak current of the conductive channel (*I_peak_*), the drain voltage (*V_D_*), and pre‐synaptic‐spike duration (*t*) according to the formula *E = V_D_ × I_peak_ × t* [31]. Therefore, reducing the energy consumption of synaptic devices requires lowering the operating voltage and current, and providing fast (time short) pulses. However, this formula should be adapted to more accurately evaluate the energy consumption of a self‐powered neuromorphic sensor, such as for neuromorphic TE‐sensors. In fact, when considering a neuromorphic memristor, a single device could be fully powered by the TE‐sensor. In tribotransistors, the gate voltage is entirely replaced by the tribopotential. As a result, energy consumption primarily arises from the need to apply a source‐drain voltage [72]. In novel ion‐gated neuromorphic transistors based on 2D‐materials semiconductors, the switching‐on potential (Vth) can be as low as a few mV, and the energy consumption drops down to few fJ. By integrating a TE‐sensor and MoS_2_‐based EG synaptic transistor, Yu et al. showed an energy consumption of only 11.9 fJ for a single‐pressure perception event [73]. The integration of TE‐sensors with high mobility transistors based on hexagonal boron nitride (h‐BN) dielectric stacked on InSe semiconductors, showing high mobility (829 cm^2^ V^−1^), allowed to reach an ultralow energy consumption down to 165 aJ/spike [74].

**FIGURE 5:**
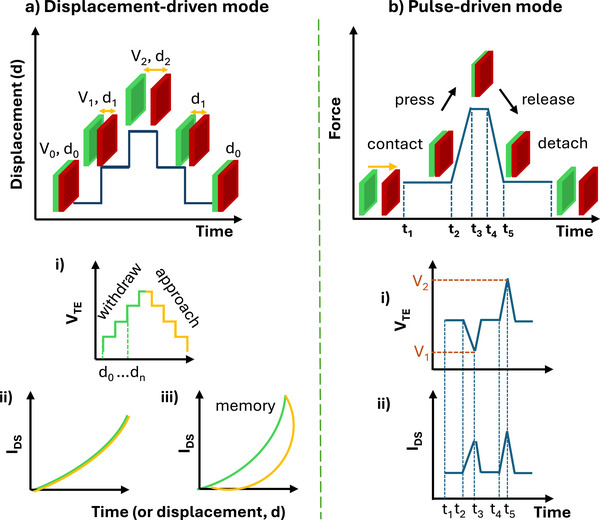
Operational modes of triboelectric neuromorphic sensors. (a) Displacement‐driven neuromorphic TE‐sensor: i) mechanical displacement dependence over time; ii‐ tribopotential dependence on displacement (D); ii) Scheme showing the stepped distance intervals (d_0_…d_n_). The increase or decrease of the tribopotential can be controlled by approaching or distancing the two triboelectrodes. The high dV_TE_/dD ensures large variation of V_TE_ with minimum distance displacement; iii) transfer curve obtained by operating a tribotransistor in displacement mode (I_DS_, source‐drain current); iv) transfer output of a tribotransistor showing memory function. (b) Pulse‐driven TE‐sensor: i) time dependence of the force applied to the TE‐sensor; ii) pulsed tribopotential originated by press and release events; iii) Example of a transistor transfer curve of a tribotransistor operated in pulse‐driven mode.

So far, two main operational modes of neuromorphic TE‐sensors have been explored, which we define here as displacement‐driven (Figure 5a) and pulse‐driven neuromorphic TE‐sensors (Figure 5b). In displacement‐driven neuromorphic TE‐sensors, the quasi‐steady tribopotential (*V_TE_*) that is established at each displacement step simulates a scanning gate bias. In the presence of memory capabilities of the neuromorphic device, the motion‐dependent transfer curve shows a hysteresis which can be exploited for multibit data storage (Figure 5a‐ii‐iii). The most used operational mode is the event/pulse‐driven mode, where touch/detach (press/release) mechanical events generate a bipolar pulsed tribopotential output that is delivered to the neuromorphic device.

Due to the time and motion‐dependent electrostatic field generated by the triboelectric charges transferred between the two friction layers, the typical TE‐sensors electrical output is characterized by an AC signal. Specifically, a single mechanical event (e.g. a contact/touch, Figure 5b‐i) generates a voltage peak that is opposite in sign to the voltage originating from the reverse mechanical event (e.g., release/detach). This spiking output is certainly a major advantage offered by TE‐sensors compared to other tactile sensors as they provide an event‐based output that is well‐suited for artificial neural network computation. However, despite this type of sensor output is more prone to emulate the event‐driven signaling sent by the human skin to the somatosensory system, the AC tribopotential shall be regulated or adapted to comply with the input requirement of artificial devices and to achieve the full TE‐sensor integration with neuromorphic devices. Additionally, the polarity of each electrode in TE‐sensors is somehow locked by the chemical nature of the tribomaterial, which is especially true in the two electrodes configuration. Therefore, while current flows back and forth through the external circuit, due to the cycling mechanical input (e.g., press/release) providing the AC output, the polarity of the triboelectrode is always the same, viz. positive charges accumulate at the electrode that is interfaced with the negative tribomaterial, and negative charges accumulate at the electrode used for the positive tribomaterials. The polarity inversion is due to the variable distance and not to the inversion of the triboelectrification field (*E_tribo_; V = E_tribo_×ds*, where *E_tribo_* does not change sign, and ds revert positive/negative during the approach and withdraw steps). Such unipolar polarization of the triboelectrode generates an electric field with one direction, which limits the ability to control both the programming and erasing operations with the same triboelectrode. So far, this problem has been overcome by 1) switching the triboelectric materials (positive and negative tribomaterials) [75, 76]. 2) switching the connection between the positive and negative triboelectrodes and the neuromorphic device input terminals; 3) designing an integrated system where the switching voltage of the neuromorphic unit (ON/OFF voltage) is tailored to suit the tribopotential variation at the triboelectrode (as in displacement driven devices coupled to transistors). While the first two solutions present an obvious technological limitation, novel device architectures have been designed to match the operating tribopotential and the switching voltage of the neuromorphic device.

**FIGURE 6:**
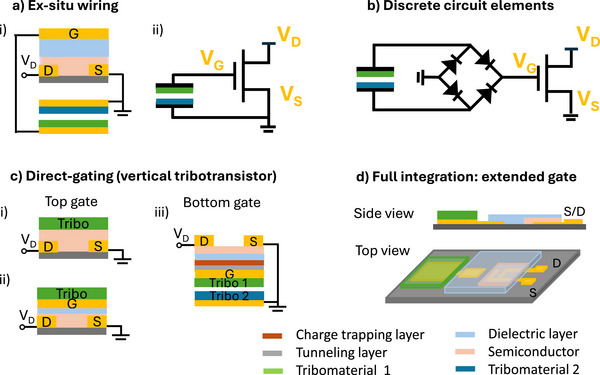
Pulse driven neuromorphic TE‐sensors architecture. (a) ex situ wiring that directly connects the TE‐sensor to a synaptic transistor through external wiring (i) and schematic equivalent circuit (ii). (b) Discrete circuits are assembled to regulate the TE‐sensor signal output priori to deliver the pulse stimuli to the neuromorphic unit. (c) Vertical tribotransistor configuration where the tribomaterial (the TE‐sensor sensing element) is integrated into the neuromorphic transistor as top gate. i) and ii) tribomaterial integrated into a top gate bottom contact transistor; iii) the triboelectrode is integrated as bottom gate into a charge trapping (floating gate) transistor. (d) Monolithic integration of a TE‐sensor and a transistor in an extended gate configuration.

According to the tribopotential regulation that might be performed (or not) before delivering the presynaptic signal to the neuromorphic device, various TE‐neuromorphic sensor architectures can be identified. Figure 6 summarizes different configurations used to integrate the TE‐sensor unit with the neuromorphic device, including: a‐ TE‐potential delivered to discrete circuit components before transmission to the neuromorphic device; b‐ ex situ wiring and direct connection to synaptic or memory devices; c‐ direct gating via vertical tribotransistors; d‐ monolithic integration as in an extended gate configuration.

In neuromorphic tribotransistors operating in contact‐separation mode, the presynaptic tribopotential (V_TE_) is transmitted to the transistor gate (presynaptic terminal) through the connection to one triboelectrode (tribomaterial interfaced to an electrode), the source electrode is set to ground along with the second triboelectrode, and the post‐synaptic signal is recorded as the source‐drain current output (I_PSC_) from the drain electrode (post‐synaptic terminal). The artificial synapses accomplish two main functions, i‐ transduce and regulate the TE‐signal by converting and decoding the tribopotential into a PSC, ii‐ rectify and amplify the presynaptic tribopotential. The PSC is dependent upon the amplitude (spatial information provided e.g., by taxel contact area and location) and width (temporal information provided e.g., by timelapse between a contact or release event, motion impulse and frequency). Mechanical programming/erasing operations can be accomplished by controlling the magnitude and polarity of the coupled V_TE_ pulses. The modulation of the channel conductance through multiple energy autonomous tactile input stimuli provides the dynamic updating of synaptic weights that carry the spatiotemporal mechanical information.

It is important to recall that in tribotransistor, while the gate bias is self‐powered, the potential provided to the drain (V_DS_, source‐drain voltage) shall be supplied by external source, which might represent the major contributing factor to the system energy consumption. In the absence of tribopotential pulses no gate‐bias is applied to the synaptic transistor, and only an off‐current contributes to the I_PCS_. Importantly, the tribopotential pulse should be high enough to trigger the transistor in the ON state under the applied source‐drain voltage (V_DS_). This implies that the overall power consumption of neuromorphic tribotransistors can be reduced by lowering the transistor threshold voltage and increasing the channel conductance, which can be achieved by using ionic dielectrics and/or high mobility semiconductors.

The operational modes introduced above naturally provide the framework for the sections that follow. Displacement‐driven and pulse‐driven modes represent the two fundamental ways in which triboelectric signals interact with neuromorphic devices. Within the pulse‐driven mode, different integration schemes, including ex situ wiring, delivery of the TE‐potential to discrete circuit components, and monolithic or direct‐gated configurations define the system‐level behavior and the types of signal transformations achievable. These architectures, in turn, underpin the neuromorphic tactile functions discussed in the next sections, such as mechanoplastic synapses, multilevel memory, nociceptive responses, and multimodal sensing. This conceptual hierarchy is used as a roadmap for the remainder of the review.

## 6 Tactile Neuromorphic Architectures

[**R1Q1**] This section focuses on tactile synaptic architectures based on neuromorphic TE‐sensors operating in displacement‐driven mode or pulse‐driven mode, where mechanical stimuli are directly transduced into synaptic‐like electrical responses. Building upon the device‐level integration strategies discussed in previous sections, the works reviewed here demonstrate how TE potential can be exploited to gate synaptic transistors and modulate synaptic plasticity through controlled mechanical displacement. Representative device architectures and operation modes are discussed, highlighting their ability to emulate key synaptic functions, learning‐forgetting behavior, and mechanosensory signal processing, thereby enabling tactile perception functionalities at the device and system level.

### 6.1 Displacement‐Driven Neuromorphic TE‐Sensor

**FIGURE 7:**
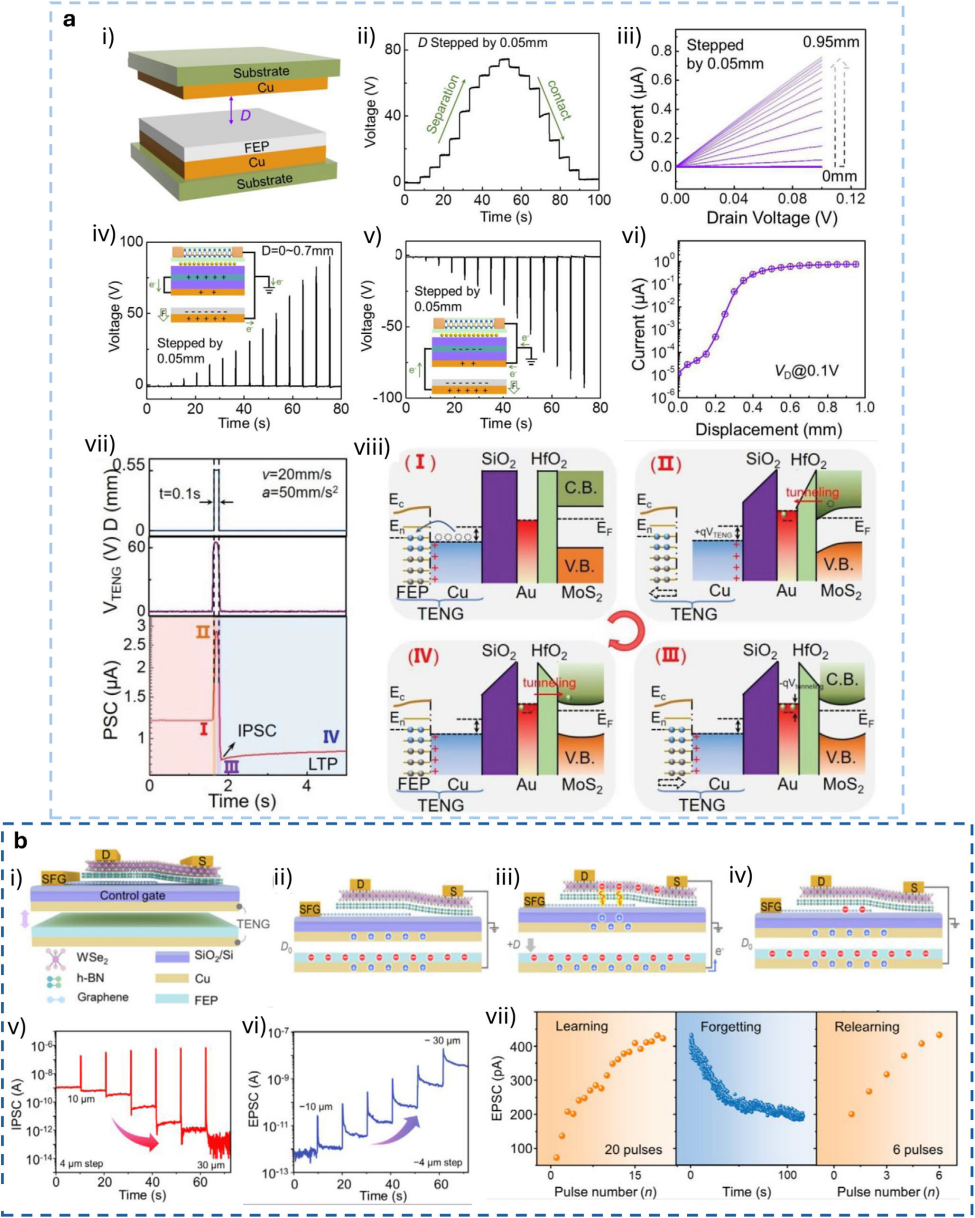
(a) i) Structure of the reference TENG used to characterize the tribopotential pulses. ii) Tribopotential voltage and iii) current variation upon displacement steps of (*D*) of 0.05 mm. iv,v) tribopotential pulses polarity inversion obtained by switching the connection between the TENG and the synaptic transistors terminals. vi) Transfer characteristic curves of a floating‐gate transistor gated by TENG (input displacement from 0 to 0.95, 0.05 mm step). vii) Mechanoplastic synaptic current originated from a single displacement pulse and the four‐stage variation of the PSC. viii) Schematic and band diagram of the operating working mechanism from Stages I to IV [77]. Reproduced with permission [77]. Copyright 2020, Wiley. (b) i) Device structure and materials components of a non‐volatile tactile tribotransistor. (ii‐iv) Schematic representation of the working mechanism during (ii) initial triboelectric charging original state, (iii) charge doping state under the application of a positive relative displacement (+D), and (iv) following the removal of the positive relative displacement. v) IPSC responses to a series of continuous D amplitudes ranging from 10 to 30 µm, (duration 0.2 s, interval 10 s). vi) EPSC responses to a series of continuous D amplitudes ranging from −10 to −30 µm, (0.2 s duration, interval 10 s), demonstrating modulated multilevel states that correspond to synaptic weight modulation. (vii) The increase in EPSC with successive D pulses represents the learning or relearning process, whereas the decay of EPSC reflects the forgetting process [78]. Reproduced with permission [78]. Copyright 2022, Elsevier.

In displacement‐driven neuromorphic TE‐sensors, after the initial triboelectrification charging that originates from the contact between the two tribomaterials, the positive and negative triboelectrodes are separated in a stepped manner, as reported in the work of Yan et al. (Figure 7) [77]. Since the tribopotential is a function of the variable electrostatic field controlled by the relative distance between the two charged triboelectric layers, a distance‐dependent electrical output is recorded at each displacement step. This step‐by‐step approach‐and‐withdraw motion (Figure 7ai‐ii) generates a discrete variation of the V_TE_. Due to the long‐lasting persistence of the triboelectrification charges on the materials surfaces (up to days in controlled conditions), at each step a quasi‐steady tribopotential is delivered to the neuromorphic device, mimicking an almost continuous voltage scan. When this stepping tribopotential is applied to the gate electrode of a transistor, the transfer characteristic can be represented with the gate bias axis replaced by a time (t) or displacement distance (d) axis (source‐drain current I_DS_ Figure 7a‐iii). The forward V_TE_ scan is then generated by a step‐by‐step re‐approach of the two tribomaterials. The authors [77]. showed a mechanoplastic artificial synapse operated in displacement mode, composed of a two‐electrode TE‐sensor and a charge trapping synaptic transistor based on MoS_2_ semiconductor (10 nm Au nanoparticles, NPs used as charge trapping layer; 10 nm HfO_2_ tunneling dielectric layer, SiO_2_ dielectric). A bottom direct gating structure (Figure 6c) is used, where the Cu triboelectrode (positive friction layer) is shared with the gate electrode. The negative triboelectrode is composed of FEP and a Cu electrode. The tribopotential of the integrated TE‐sensor can be modulated from 0 to ∼ 75 V through step‐by‐step displacement variation (0.05 mm steps, Figure 7a‐iv,v), which translates into a stepped positive potential applied to the Cu electrode shared with the synaptic transistor. The authors also showed PPF and PPD behavior by a manual swap of the TE‐sensor synaptic device connection (see Figure 7a‐iv,v), showing how switching the connection between the TE‐sensor and the synaptic transistors corresponds to inverting the polarity of the tribopotential pulse sent to the gate. The transfer curves reported in Figure 7a‐vi clearly show how the displacement (i.e., the V_TE_) is effective in controlling the gate bias modulation (V_DS_ = 0.1 V). The PSC showed a mechanical pulse‐dependent dynamic where four different stages could be identified in the PSC output (Figure 7a‐vii), which have been explained according to the energy diagram shown in Figure 7a‐viii. Stage I corresponds to the contact conditions where no displacement occurs, and the I_PSC_ is stable (steady state), and interfacial triboelectric charges accumulated at the positive and negative triboelectrodes leave unaffected the channel conductance (V_G_ = 0 V). Upon displacement (stage II) a tribopotential pulse triggers the gate up to 60 V with a simultaneous increase in I_PSC_. The positive tribopotential at the gate causes the injection of negative charges at the source, part of which tunnels to the Au NPs layer, where they are trapped. The restoration of the initial contact condition (stage III) brings back the tribopotential to 0 V, but the presence of the trapped negative charges at the floating gate establishes a time persistent negative gate voltage inducing an inhibitory PSC. In stage IV, the trapped charges recombine flowing back through the tunneling layer and the PSC decay back to the steady state (back to stage I). The retention time is longer than seconds denoting the presence of synaptic plasticity allowing to implement a mechano‐switching logic converter. The synaptic weight is not only dependent on displacement distance but also on the duration time of stage II (stepping speed 20 mm/s).

Jia et al. [78] showed a direct gated reconfigurable tribotronic nonvolatile system operated in displacement mode. The device is composed of a vdW heterostructure comprising a stacked heterostructure of graphene/hexagonal boron nitride (h‐BN)/tungsten diselenide (WSe_2_), where charge carriers trapping and de‐trapping occur in the graphene semifloating‐gate layer (Figure 7b‐i). The author reported an energy consumption of 74.2 fJ per synaptic event (spike). The type and density of charge carriers in the semifloating layer, can be flexibly tuned and reversibly switched, thus allowing the channel conductance to exhibit nonvolatile and continuously tunable behavior (Figure 7b‐ii‐iv), allowing short‐term and long‐term plasticity. Moreover, the synaptic response is sensitive to the amplitude and duration of external mechanical stimuli. Figure 7b‐v, shows how an increase in mechanical displacement (from 10 to 30 µm, 0.2 s step‐duration), causes the IPSC to decrease stepwise, creating multilevel synaptic states. Similarly, after applying a positive triboelectric potential under reverse displacements (−10 to −30 µm), the EPSC shows a stable stepped increase (Figure 7b‐vi). Learning/forgetting behavior is demonstrated using two sequences of D pulses (Figure 7b‐vii). During the first 20 pulses, EPSC gradually increases, indicating a learning process. After pulse removal, EPSC decays over 120 s, representing forgetting. In the second stage, only 6 pulses are needed to restore the same current, mimicking relearning and suggesting that previously learned information is retained. Incorporating a charge trapping layer facilitates the mechanical modulation of synaptic weights, enabling the implementation of both STP and LTP within a single device. In a different work, Jia et al. [79], presented a multibit non‐volatile memory based on displacement‐driven and direct‐gated tribotransistor. PTFE and Cu were used as a positive triboelectrode and another Cu layer as a negative triboelectrode. The positive triboelectrode was integrated into a Si‐SiO_2_ gated transistor comprising a van der Waals heterostructure including MoS_2_ as a semiconductor, graphene as a floating gate, and h‐BN as a tunneling dielectric layer. The authors reported a high on/off ratio of 10^5^ achieved with a mechanical displacement of 0.4 mm, 6000 s of retention time, and multilevel data storage (14 stages achieved at different displacements). The integration of a load resistor at the drain electrode enables the inverter function, thus showing the possibility to transduce the tribopotential into logic signals.

### 6.2 Pulse‐Driven Neuromorphic TE‐Sensor

#### 6.2.1 Ex Situ Wiring and Direct Connection to Synaptic or Memory Devices

**FIGURE 8:**
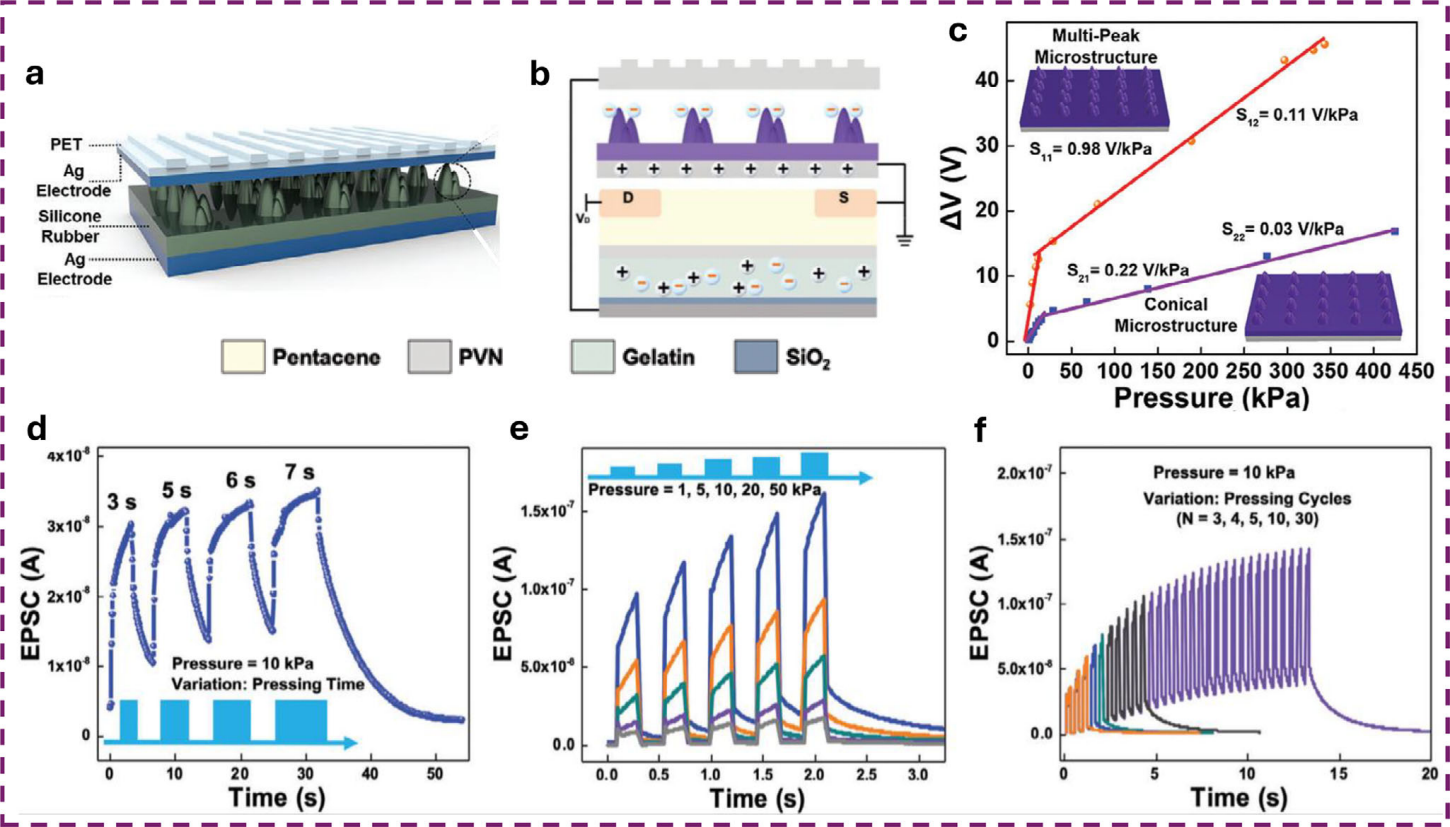
(a) Schematic structure of the TE sensor and (b) of the organic synaptic EDLT. (c) Sensitivity of the TE‐sensor, including or not the multi‐peak microstructures. (d) Time‐dependent EPSC triggered by the same force load (10 kPa) but different static pressure time durations. (e) EPSC dependence on pulses of variable pressure load but the same duration. (f) Dependence of the EPSC on the number of train pulses at a constant force of 10 kPa, showing PPF features [82]. Reproduced with permission [82]. Copyright 2024, Wiley.

The most basic synaptic tribotransistor architectures rely on an ex situ connection, where the TE‐sensor is externally connected to the synaptic transistor without direct physical integration [80, 81, 82]. Typically, the TE‐sensor is connected to the synaptic transistor by directly wiring one of its triboelectrodes to the gate terminal, and the other to the source terminal, which is held at ground potential. The most common transistor configuration is the bottom‐gate top‐contact, where the bottom gate electrode and dielectric layer are Si and SiO_2_, respectively. Lei et al. presented an artificial tactile near sensor based on a TE‐sensor and an EDLT (Si‐SiO_2_ bottom gate, gelatine ionic dielectric, pentacene semiconductor, Figure 8a,b) [82]. The presence of multipeak microstructures in the TE‐sensor unit (Figure 8a) allowed to reach a sensitivity of 0.98 V kPa^−1^ over 0–10 kPa, and 0.11 V kPa^−1^ up to 350 kPa (Figure 8c). By introducing a top surface patterned with stripes and soft features on the back of one triboelectrode, the authors showed a bimodal perception where both sliding and pressure sensing could be achieved. A clear dependence of the excitatory post‐synaptic current (EPSC) on pressure, pulse duration, and number of pulses is shown in Figure 8d–f respectively. The increasing EPSC with the number of pulses demonstrated the PPF, thus showing the effectiveness of a STP of the neuromorphic tactile sensor (Figure 8f).

Due to their high mobility, semiconductors based on 2D‐materials have often been integrated into ion‐gated synaptic transistors for neuromorphic TE‐sensors. Xie et al., reported on an all‐solid‐state synaptic EDLT based on Mxenes and AlO_x_‐Li as a solid‐state ionic dielectric [51]. To simulate static and dynamic pressure, the authors fabricated an integrated triboelectric‐and‐capacitive sensor, capable of sensing the dynamic touch from the electrification of a triboelectric layer and the static pressure by means of a capacitor array.

#### 6.2.2 TE‐Potential Delivered to Discrete Circuit Components

**FIGURE 9:**
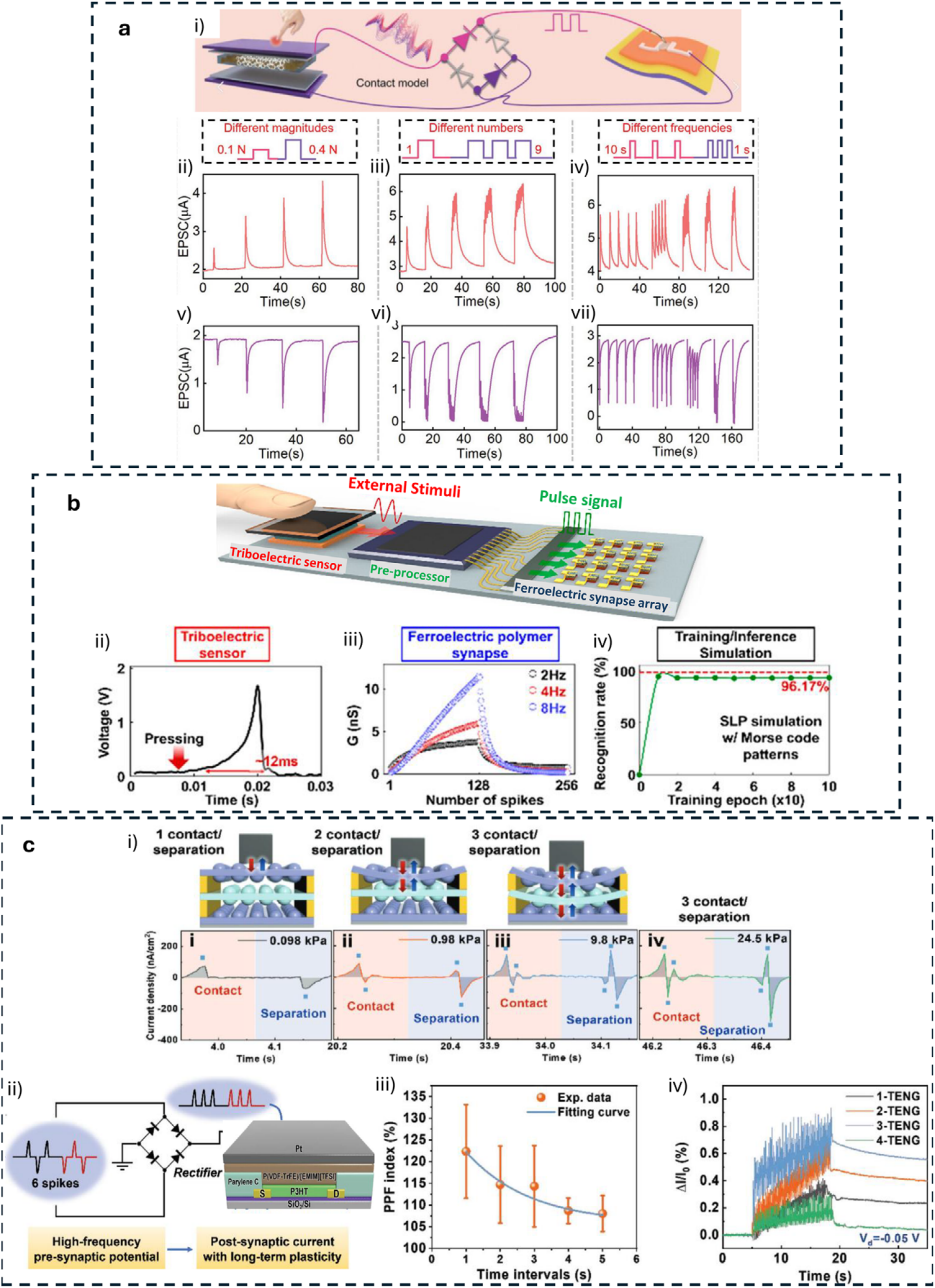
(a) i) Scheme of a TE‐sensor based on a NiCo_2_S_4_/S‐polymethyl‐methacrylate composite bridged through a full‐wave rectifier to a charge‐trapping In_2_O_3_/IGZO synaptic transistor. ii‐iv) EPSC response to positive tribopotential pulses varying pressure load (ii), number of external stimuli (iii), and mechanical stimulus frequency (iv). V‐vii) IPSC response to negative tribopotential pulses [83]. Reproduced with permission [83]. Copyright 2024, Wiley. (b) i) Scheme of a tactile neuromorphic system composed of a TE‐sensor, a microcontroller unit (processor), and an array of ferroelectric synaptic transistors. ii) Real‐time tribopotential generated by a touch stimulus. iii) Synaptic LTP and LTD measured as conductance (G) time dependence at varying pulse frequency. iv) Recognition rate as a function of the training epoch for the single‐layer perceptron‐based neural network using Moore code alphabets [85]. Reproduced with permission [85]. Copyright 2023, ACS. (c) i) A 3‐layer‐TENG device capable of generating two, four, and six spikes according to the applied pressure load during a single touch event. ii) Scheme of the pre‐synaptic potential sent through a full‐wave bridge rectifier to the synaptic OECT. iii) PPF index showing the STP. iv) EPSC recorded for TENGs comprising a different number of layers, showing the best performance obtained for the 3‐TENG [86]. Reproduced under terms of the CC‐BY license [86]. Copyright 2023, Wiley.

Another important aspect of efficiently integrating the TE‐sensor into neuromorphic devices is fully utilizing the information from the positive and negative tribopotential peaks associated to the contact‐and‐release event. Since the direction of the triboelectrification field remains constant during mechanical cycles, achieving better control over the program and erasing operations in neuromorphic devices requires control over the polarity of the tribopotential pulses. To this aim, the TE‐sensor AC signal can be rectified with discrete circuit components based on conventional silicon electronics, as rectifying bridges and logic circuits, before being sent to the neuromorphic unit. This approach is typically used for integration with 2‐terminal devices, where reversing the polarization of the tribopotential is necessary to trigger the reset process (e.g. filament breaking) required to enable write‐and‐erase memory function. The simplest circuit used to interface the TE‐sensor with the neuromorphic device is a full‐wave rectifier bridge, which rectifies the AC tribopotential into unipolar voltage peaks. By selecting which terminal of the rectifier is connected to ground, the polarity of the tribopotential delivered to the base electrode of a memristor or to the gate of a transistor can be effectively controlled. Qi et al. [83] employed the rectified positive tribopotential to trigger EPSCs as shown by the increase in current under a train of tribopotential pulses (Figure 9a‐ii–iv). Conversely, the negative tribopotential was used to induce inhibitory post‐synaptic currents (IPSCs), resulting in a current decrease under similar pulse conditions (Figure 9a‐v–vii). Fang et al. connected a TENG to a FeFET through a bridge rectifier circuit [84], realizing a memory device where the writing and erasing process could be achieved by finger tapping and upon switching the connections to either the positive or negative terminal of the rectifier, respectively (V_DS_ = 8 V).

Neuromorphic processors and more complex logic circuits have also been used to produce multiple spikes from single touch‐detach events and to more closely mimic biological functions. In fact, per each tactile sensing event, and depending on its specific amplitude and dynamics, the afferent nerve can generate multiple action potential pulses, which are then processed in the somatosensory cortex. The decomposition of a sensing event into multiple spikes that are then digitalized is also at the base of efficient neuromorphic computing in SNN. To emulate such spiking behavior, Kim et al. [85]. interfaced a TE‐sensor based on polydimethylsiloxane (PDMS) with a hardware neural network processor capable of delivering a pulsed signal to an array of ferroelectric synaptic transistors (MoS_2_ semiconductor, poly(vinylidene fluoride), PVDF, as ferroelectric layer) (Figure 9b‐i). The processor has the role of generating multiple pulses from a single event‐triggered analog signal (e.g., press or release), extracting 400 voltage values from a single tribopotential pulse. The spiking voltage pulses are then fed to an array of 20 × 20 ferroelectric synaptic transistors. The synaptic array output showed LTP and LTD behaviors, and the system was used to conduct training and recognition simulations using Moore code alphabets and MNIST handwritten digits, achieving a recognition rate of 96.17 % (Figure 9b‐ii‐iv).

To maintain the advantage of self‐powered electrical inputs while also providing enough pulses to enable efficient neuromorphic computation, Park et al. [86] fabricated a multilayered TENG capable of delivering multiple tribopotential pulses following a single touch event (Figure 9c). This approach offers the advantage of enhanced LTP, as the multilayered structure can generate multiple tribopotential pulses (2–4 per event) from a single touch, mimicking high‐frequency stimulation. This effect is typically achieved in single‐layer TENGs only under continuous mechanical input at frequencies exceeding 10 Hz. The proposed approach more closely emulates the human tactile sensing, where for a single tactile event, the afferent nerve generates multiple action potential pulses (Figure 9c‐i). The multilayer TENG is composed of micro‐patterned films based on PDMS‐BaTiO_3_ (BTO) composite that is functionalized with fluoroalkyl silane used to increase the film negative triboelectric charge density. The multilayer‐TENG was interfaced through a full‐wave rectifier bridge to a synaptic organic electrochemical transistor (polythiophene semiconductor and PVDF‐TrFE (poly(vinylidene fluoride)‐co‐trifluoroethylene) based polyelectrolyte, Figure 9c‐ii). Repeated mechanical stimulations enabled a transition from STP to LTP with high synaptic weight (Figure 9c‐iii‐iv). The integrated neuromorphic sensor was used to control a robotic hand, being capable of simulating memory, training, and associative learning.

Liu et al. [76]. presented an artificial sensory memory system based on a 28 × 28 triboelectric sensory array designed for real‐time neuromorphic computing, which enabled real‐time recognition of handwritten images and supports large‐scale data processing. The TE‐sensor included two structured friction layers: a nano‐structured copper (Cu) as a positive triboelectrode and a micro‐structured PDMS‐coated Cu electrode serving as the negative triboelectrode. The sensor exhibited a linear pressure sensitivity of 0.192 kPa^−^^1^ in the low‐pressure range (∼0.68 Pa–∼5 kPa), and 0.007 kPa^−^^1^ in the higher‐pressure range, showing a saturation‐like force response (response times 3–8 ms). The sensor matrix is constructed as a crossbar electrode array, with the interlayer formed by uniformly micro‐structured PDMS. Artificial synapses were connected ex situ and were based on ionic liquid dielectrics (LiTSFI)‐based synapse, a DPP‐DTT organic semiconductor, gold (Au) electrodes and a Si/SiO_2_ gate. Synaptic weights were modulated by varying external pressures. Recognition is achieved by feeding voltage outputs from each electrode into a pretrained neural network, enabling handwritten character identification. Initial neural network training was performed using data from a single TENG sensor, connected ex situ to a synaptic transistor.

#### 6.2.3 Monolithic Integration and Direct Gating

Although heterogeneous integration has been widely explored, monolithic integration of TENGs with synaptic devices offers a more biomimetic architecture, facilitating direct energy coupling, minimizing interfacial resistance, and significantly reducing energy dissipation. TE‐sensors have been integrated into neuromorphic tribotransistors to form a monolithic structure and to avoid the necessity of external wiring between two units. In this configuration, the active layer of the TE‐sensor, viz. the tribomaterial, can be integrated in two main ways: 1‐ as an integrated triboelectrode positioned at the gate interface, enabling direct gating in either top‐gated or bottom‐gated transistor architectures; or 2‐ by delivering the tribopotential to the gate electrode through a shared dielectric medium, typically an ionic dielectric, in what is known as an extended gate configuration. The latest configuration is still very poorly explored, while the most typical configuration is the bottom‐gate top‐contact due to the often‐optimized Si/SiO_2_ gated synaptic transistors.

**FIGURE 10:**
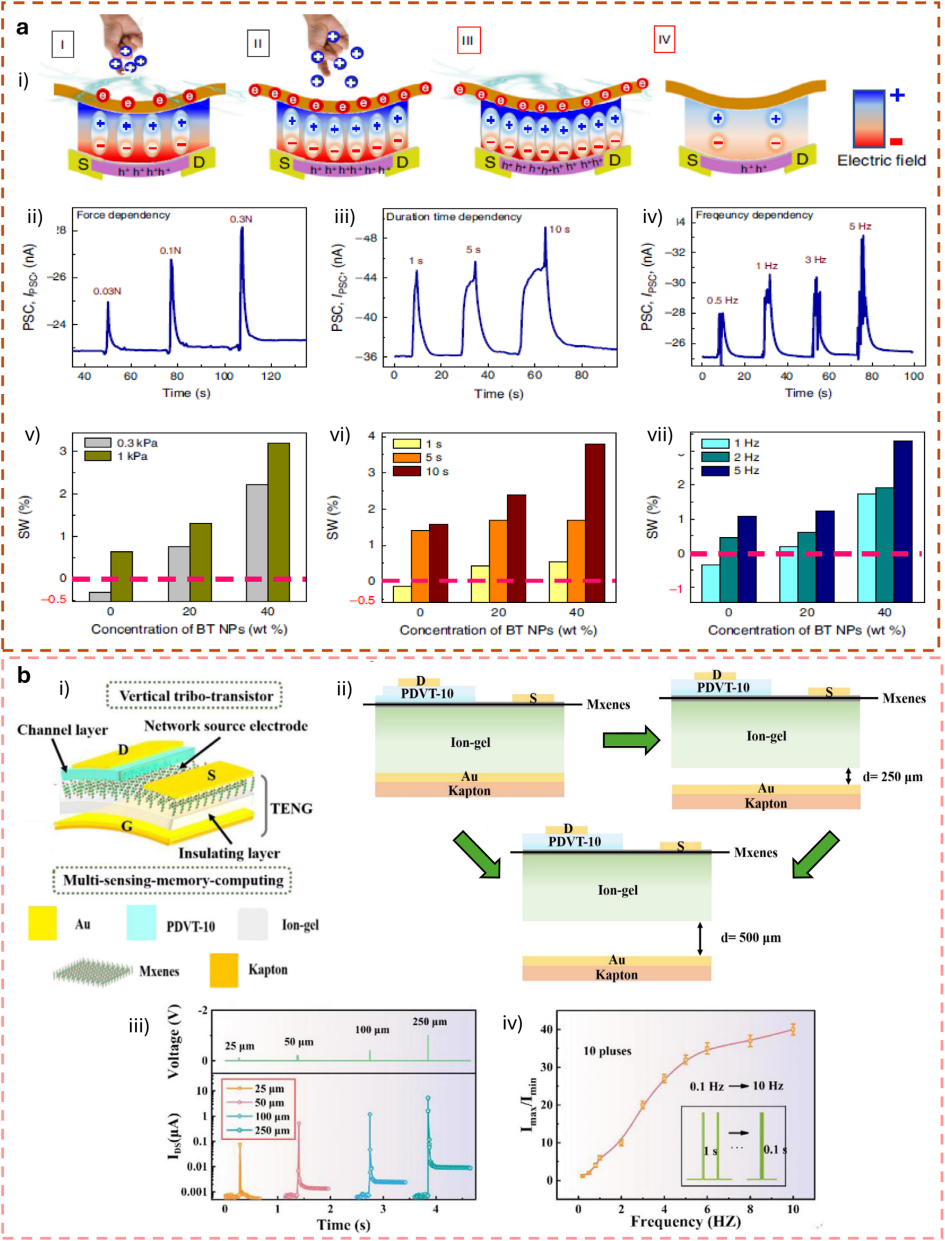
(a) i) Device structure and schematic mechanism of the tribopotential triggered dipole switching in the ferroelectric layer (I and II dipole alignment and holes accumulation in the channel induced by tactile stimulation and III‐IV ferroelectric dipole relaxation and hole current decrease). PSC (V_DS_ –3 V) recorded during a tactile stimulus at varying ii) pressures, iii) duration (≈1 kPa), and iv) rate (≈1 kPa for 5 s). SW relative changes for different compositions of the ferroelectric nanocomposite under varying v) pressure, vi) durations (≈1 kPa), and vii) frequencies of touch (≈1 kPa). The authors defined an arbitrary SW threshold (red dotted line) for simulating noise filtering in biological mechanoreceptors [87]. Reproduced under terms of the CC‐BY license [87]. Copyright 2020, Springer Nature. (b) i) Schematic illustration of the structure of the direct gated vertical tribotransistors. ii) Working mechanism of the sensor operating in displacement mode. iii) EPSC recorder at different distance and iv) I_max_/I_min_, dependence on pulse frequencies (I_max_ is the 10th EPSC amplitude, I_min_ is the first EPSC amplitude) [88]. Reproduced under terms of the CC‐BY license [88]. Copyright 2022, Springer Nature.

A triboelectric‐capacitive coupled tactile sensor based on ferroelectric organic field‐effect transistors (Fe‐OFET) emulating Merkel cell function was presented by Lee et al. [87]. The sensor included an organic synaptic transistor with a BaTiO_2_/PVDF‐TrFe ferroelectric composite used as gate dielectric, pentacene as semiconductor, and a polyimide film deposited on the composite that acted as tribomaterial. The ferroelectric dipole switching under tactile pressure facilitated slow adaptation. When a negative tribopotential is applied to the gate, dipoles in the ferroelectric layer align downward (Figure 10a‐i), increasing carrier accumulation and drain current. When the tactile stimulus is removed, polarization slowly relaxes back to its previous orientation, mimicking a memory process, with channel conductance restoration analogous to forgetting (Figure 10a‐i). The weight of the synaptic response varies with touch force, duration, and frequency (Figure 10a‐ii–iv, respectively), with a transition from STP to LTP arbitrarily defined at forces above 3 kPa. The synaptic weight was dependent on the ferroelectric layer composition, allowing tuning the threshold for filtering and memory functions (Figure 10a‐v‐vii).

Khan et al. [75] used a PDMS layer as a top‐gating material and dielectric layer interfaced with a MoS_2_ semiconductor channel. While the memory function was triggered by direct contact to the top tribomaterial, the device was also allowing external bias gating to a bottom Si/SiO_2_ back gate. According to the electron affinity of the material in contact with the sensor tribomaterial (mechanoreceptor side of the tribotransistor) a current increase or decrease was observed. Due to the slow triboelectric charge dissipation, after removal of the tactile stimulus, the I_PSC_ decreases slowly, lasting from minutes to few hours, according to the selected material. In particular, the long‐lasting triboelectric charges trapped in the PDMS layer following direct contact enable a zero‐power write operation for touch memory, accompanied by a threshold voltage shift that depends on the nature of the contacting material.

An integrated configuration was proposed by Liu et al. [88], avoiding redundant layers. Specifically, a MXenes layer and the source electrode are common elements shared between the triboelectrode (staked structure Au/MXenes/ion gel, where the ion gel also works as tribomaterial) and the vertical organic EGT (Figure 10b‐i). The second triboelectrode is composed of an Au‐coated Kapton film that is also acting as a mobile gate electrode. Due to the short channel length corresponding to the thickness of the PVDT‐10 semiconductor layer (a few nm), a large channel conductance variation was observed upon operation in displacement‐driven mode (Figure 10b‐ii), and the signal amplification operated by the transistor allowed a 711‐time increase in sensitivity to the triboelectrodes relative distance. The neuromorphic tactile response was recorded at varying the gate voltage under controlled variation of the distance between the gate electrode and the ion gel tribomaterial, and constant V_DS_ (−0.5 V). Synaptic plasticity was associated with the presence of the ion gel and its slow ionic transport. Under increased displacement from 25 to 250 µm, the equivalent gate voltage varied from 0.5 to 1.5 V, and the I_PSC_ rose from 0.1 to 8 µA (Figure 10‐iii). By varying the frequency from 1 to 10 Hz, the relative current increased from a factor 1.2 to 40 (Figure 10‐iv). Additionally, the possibility of exploiting both sound and light sensitivity of the device allowed for a multi‐sensing neuromorphic computing, which was further employed to demonstrate a multi‐model emotional recognition.

**FIGURE 11:**
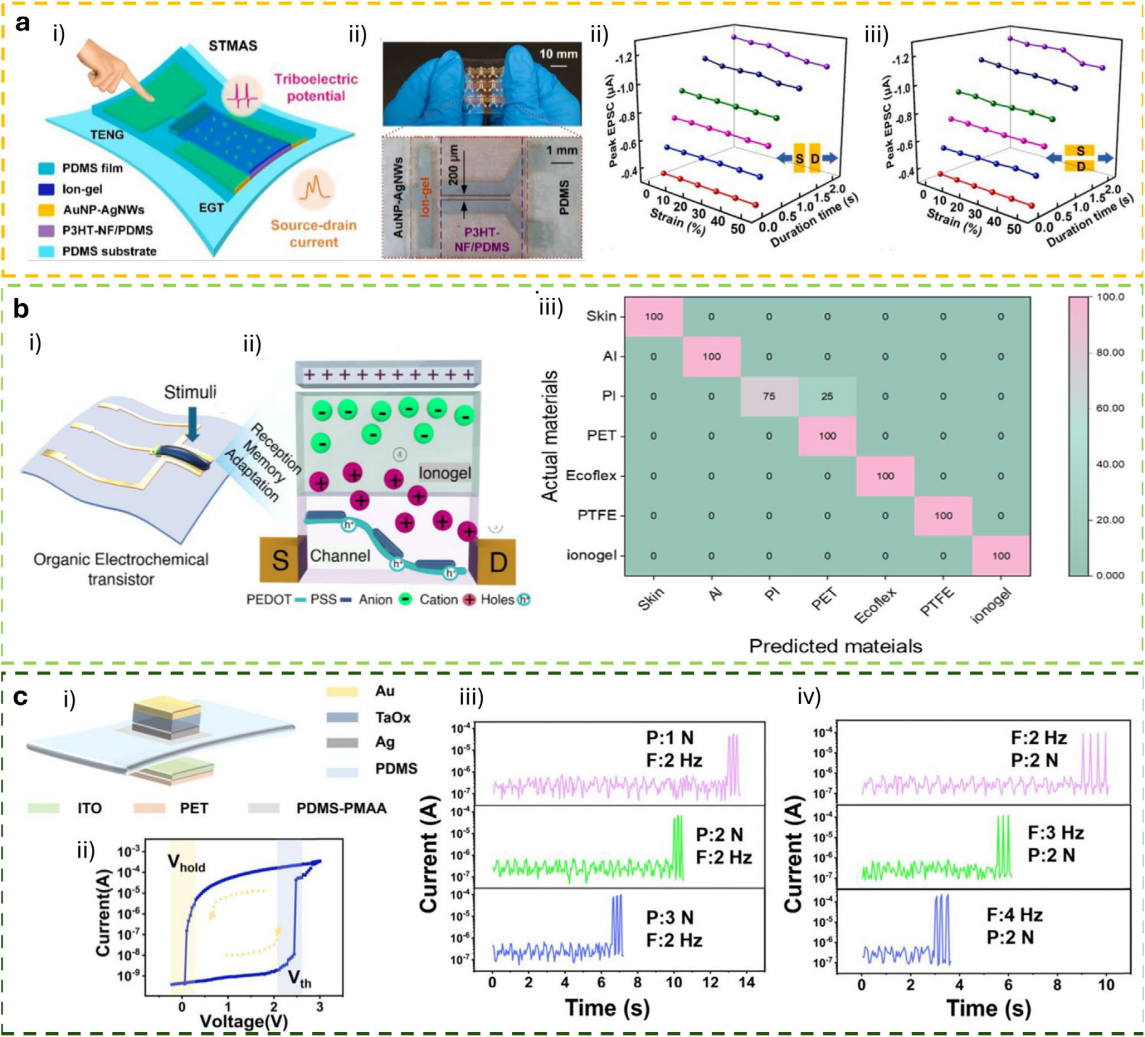
Monolithic neuromorphic mechanoreceptors based on flexible and stretchable neuromorphic devices. (a) i) Schematic picture of the stretchable and fully integrated mechanoplastic synapse and ii) optical photograph of the stretchable synapse. Synaptic plasticity under different stretch strain: iii) EPSC peak values dependence on tribo‐pulse duration and strain applied along a direction parallel to the transistor channel direction and (iv) perpendicularly to the channel [90]. Reproduced with permission [90]. Copyright 2024, Elsevier. (b) i) Schematic structure of the monolithic organic mechanoreceptors where the ion gel dielectric (blue over the transistor channel) is also acting as a tribomaterial. ii) schematic representation of the ionic drift triggered by contact with a positive tribomaterial (cations move toward the PEDOT:PSS semiconductor channel, anions move toward the top side of the ion gel film). iii) Machine learning algorithm generated a confusion matrix showing materials recognition and the classification accuracy per each material [91]. Reproduced with permission [91]. Copyright 2024, Elsevier. (c) i) Structure of the stretch insensitive neuromorphic tactile sensor. ii) Threshold switching performance of the TaO_x_‐based neuron. Neuron Firing time dependence on iii) pressure load and iv) frequency applied to the TE‐sensor [93]. Reproduced with permission [93]. Copyright 2024, RCS.

To advance the development of biocompatible and conformable neuromorphic devices, it is essential to establish fabrication processes based on materials capable of withstanding elastic deformation, while also overcoming the inherent limitations of conventional silicon‐based electronics [89]. Monolithic integration of organic neuromorphic devices with TE‐sensors on flexible substrate is often achieved by using and extended gate and planar configuration. Zeng et al. [90] presented a fully integrated system based on intrinsically stretchable mechanoplastic artificial synapses, based on an extended‐gate electrolyte‐gated organic‐FET (EGOFET) and a TE‐sensor. The two units share a common flexible substrate, and a stretchable floating gate electrode based on Ag‐nanowires (AgNWs)/PDMS composite. The PDMS is also used as a negative triboelectric layer. An ion gel dielectric layer made of a solid electrolyte polymer dielectric poly(vinylidene fluoride‐co‐hexafluoropropylene (PVDF‐HFP) and the ionic liquid 1‐ethyl‐3‐methylimidazolium bis(trifluoromethylsulfonyl)imide (EMIM‐TSFI) was deposited onto a poly(3‐hexylthiophene) (P3HT) nanofiber stretchable composite, which served as a semiconductor (Figure 11a‐i). A Cu layer is used as a grounded mobile positive triboelectrode, and the device was operated in displacement mode. The authors showed a stable device response under different plastic deformation (from 0% to 50% strain, up to 200 cycles) applied along the direction parallel or perpendicular to the transistor channel (Figure 11a), indicating that the elastic deformation has a relatively small impact on synaptic plasticity.

In their report, Sultan et al. [91] showed the direct coupling of the TE‐sensor and synaptic organic electrochemical transistor (OECT), where the ion gel served as both the gate dielectric and mechano‐responsive triboelectric layer (Figure 11b‐i‐ii). The ion gel was based on an ionic liquid and fluorinated polymer dielectric composite (PVDF‐HFP and EMIM‐TSFI ionic liquid), while poly(3,4‐ethylenedioxythiophene) polystyrene sulfonate (PEDOT:PSS) was used in the transistor channel (area 5 × 1 mm^2^). The depletion mode OECT device operated in a normally “on” state (*V_DS_* 0.5 V) due to the presence of mobile charge carriers in their natural on state. The authors showed slow adaptation of synaptic function and material recognition through machine learning techniques. The synaptic organic electrochemical tribotransistor showed spike number‐dependent plasticity (SNDP), spike amplitude(pressure)‐dependent plasticity (SADP), and spike duration‐dependent plasticity (SDDP), along with STP and LTP. The raw generated signals were preprocessed before computational analysis for materials recognition using machine learning. Figure 11b‐iii shows the confusion matrix identifying different materials and showing a 97% classification accuracy.

A complete circuit printed system comprising a TE‐sensor, rectifier diodes bridge, and organic transistors (P3HT nanofibers as semiconductor, and Ag NWs as electrode) on a flexible substrate was presented by Shim et al. [92] The system could convert the presynaptic TE‐sensor unit signal into a post‐synaptic rectified I_PSC_.

Liu et al. [93] integrated into a single device a stretch‐insensitive TE‐sensor and TaO_x_‐based memristor as artificial neurons, aiming to develop tactile neuromorphic sensors with skin‐like properties and maintaining stabile sensing during deformation and stretching. An ITO/polyethylene (PET) layer is used as mobile electrode (Figure 11c‐i) while the memristor and the TE‐sensor shared the same Ag‐based electrode. The formation of Ag‐conductive filament into the TaO_x_ dielectric caused the switch ON/OFF of the HRS and LRS for the memristor [94], Figure 11c‐ii). The threshold voltage‐dependent integrate‐and‐fire properties of the neuron under varying tactile pressure and frequency are presented in Figure 11c‐iii‐iv. A 64 × 64 neuromorphic tactile matrix was used to simulate the pressure trajectory and texture of a tactile perception based on firing time classification.

## 7 Neuromorphic TE‐Sensors for Advanced Tactile Sensing Applications

While the approaches discussed above mainly focus on the integration strategies and operational principles of individual neuromorphic TE‐sensors, their full potential emerges when these devices are organized into higher‐level tactile synaptic architectures capable of spatiotemporal signal processing and perception. In the following section, we therefore move from device‐level concepts to system‐level tactile synaptic architectures and their emerging applications.

### 7.1 Memory (Multibit Storage) Function and Integrated Neurons

Compared to memristive devices, synaptic devices based on neuromorphic transistors suffer the limits of poorer information filtering and “reliable coding.” Devices with inner threshold switching, such as memristors, might overcome the problem of information redundancy and energy consumption due to data storage and are often found in integrated tactile neurons. Memristors are more prone to be integrated into applications where a continuous synaptic weight update is not necessary, as there is no need to retrieve continuous stimulus information [72]. Memristors bear a history‐dependent resistance and an electrical stimuli‐dependent threshold. Non‐volatile memristors are largely investigated as a synaptic element for in‐memory computing applications, while diffusive memristors only retain state when potential is applied, therefore, they show a volatile memory controlled by threshold switching [72].

**FIGURE 12:**
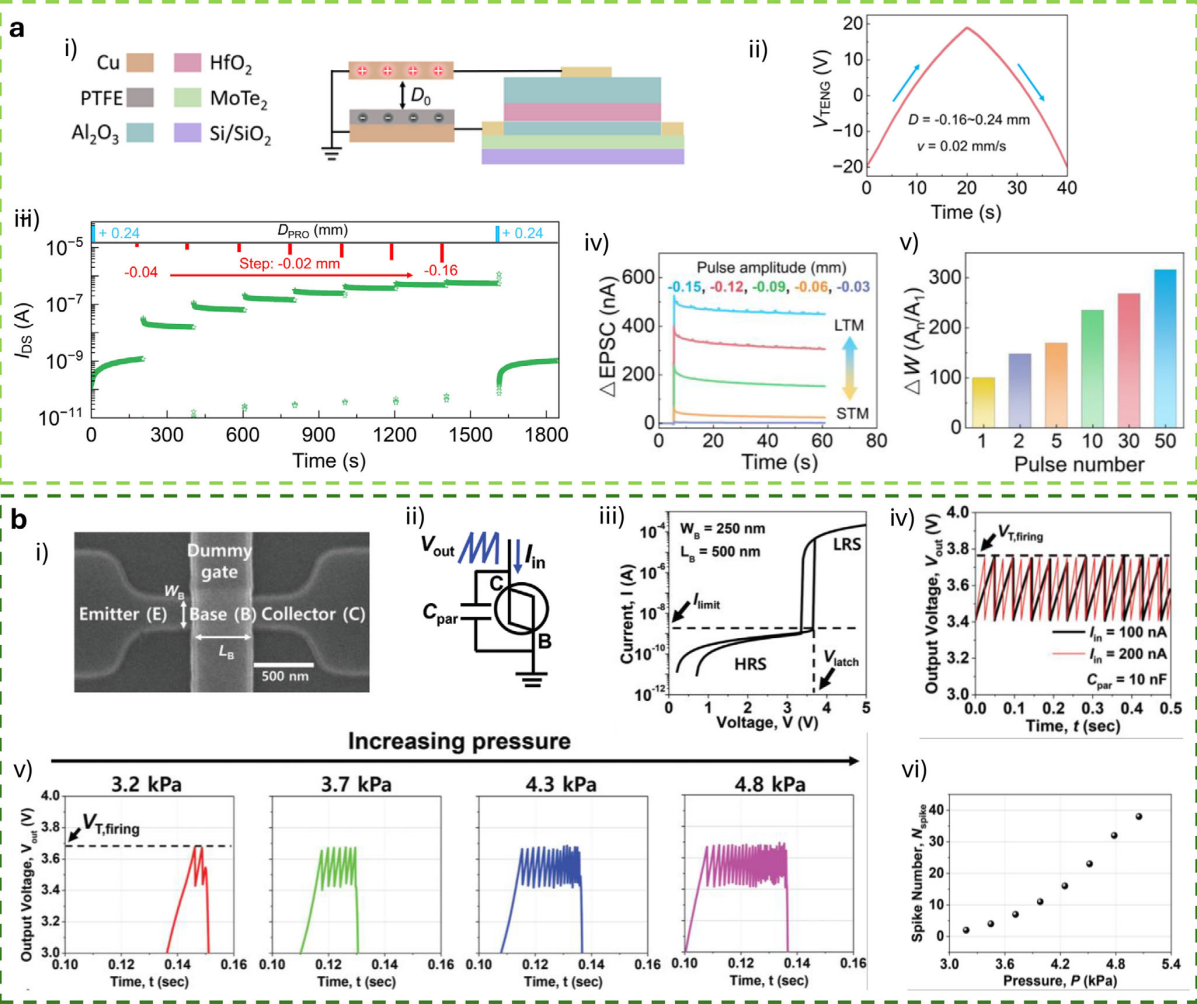
(a) Mechano‐driven logic‐in‐memory: i‐ Scheme of the neuromorphic triboelectric charge‐trapping transistor. ii‐ Tribopotential modulation during approach and withdrawal motion operated at constant speed. iii‐ Sequence of Program‐Read‐Erase‐Read following different displacements (*D*_PRO_, ranging from −0.04 to −0.16 mm, pulse width 0.5 s, reading at D = 0.24 mm) used for programming and showing dynamic memory properties. iv‐ EPSCs recorded by applying several displacement pulses with different amplitudes (fixed pulse width of 0.5 s). v‐ The synaptic weight dependance on pulse number (ΔW= A_n_/A_1_, *V*_DS_= 30 mV) [26]. Reproduced under terms of the CC‐BY license [26]. Copyright 2024, Elsevier. (b) Multi‐spikes encoding of a single touch event i) SEM image of the fabricated biristor neuron. ii) Equivalent circuit of the pulsed integrate‐and‐fire neuron based on the BJT (biristor), iii) its current–voltage characteristics (latch‐up voltage, output voltage recorded at the capacitor), and iv) the time‐dependent voltage output spiking characteristics. v) Time‐dependent voltage spiking characteristics at varying applied pressures. vi) Number of spikes generated during a single touch as a function of pressure [95]. Reproduced under terms of the CC‐BY license [95]. Copyright 2022, Wiley.

Spiking encoding is better performed by artificial synapses based on 3‐terminal devices, while filtering is better performed by 2‐terminal devices. An optimal artificial tactile sensor would combine both functions to ensure a real‐time processing of sensory data in recognition tasks. Wei et al. [26] showed that mechano‐driven logic‐in‐memory was achieved with a neuromorphic triboelectric charge‐trapping MoTe_2_ transistor. The semiconductor charge carriers (electrons or holes) tunnel through the Al_2_O_3_ tunneling barrier layer and are stored in the HfO_2_ charge‐trapping layer (Figure 12a‐i). The device was operated in displacement mode. The logic function was achieved by integrating a load resistor in series to the source‐drain channel, realizing a mechano‐driven inverter like an *n*‐type MOSFET resistors‐transistor logic inverter. The system enabled the demonstration of a prototype integrating perception, storage, and logic/computing functionalities. Key performance metrics included a switching ratio greater than 10^5^, a program/erase ratio > 3 × 10^2^, a low off‐state current of about 0.6 pA, a memory retention time of up to 10^4^ s, a multilevel data storage of 8 levels (Figure 12a‐iii), and operational stability maintained for up to 3 months. The author reported EPSC, PPF, STM, LTM, and learning forgetting behaviors simulated under mechanical displacement pulse (Figure 12a‐iv‐v). The integration of the synaptic tribotransistor with a 3‐layers perceptron ANN algorithm for pattern recognition using the MNIST (Modified National Institute of Standards and Technology) dataset allowed mechano‐driven recognition of handwritten digits, achieving an accuracy of approximately 88.6%.

In terms of energy consumption, the advantage of using SNN algorithms becomes more tangible when increasing the number of input data to compute. However, when dealing with the low‐frequency tactile inputs, or in the event of a single touch the advantage of using SNN to save energy might be limited. By using a bipolar junction transistor (BJT, also called biristor, Figure 12b‐i, with a single‐transistor latch (STL) structure, Han et al. [95] demonstrated a fully self‐powered tactile spiking neuron capable of encoding the applied force through the generation of an output characterized by multiple voltage spikes per mechanical event. The BJT withstands an HRS and LRS when the current limit is below or above a certain threshold (Figure 12b‐iii‐iv) and can convert the TENG input current into a spiking voltage output, similarly to a spiking neuron which can fire multiple output spikes from a single input signal. The current output of the TE‐sensor (2‐electrode configuration, Al as positive triboelectrode and PTFE as negative tribomaterial, 4 × 4 cm^2^) is delivered to the collector (*n*‐type semiconductor). This induces hole injection from the base into the p‐semiconductor, while electrons move from the emitter (*n*‐type semiconductor, grounded electrode) toward the floating base electrode, generating electron‐hole pairs within the junction. A leaky integrate‐and‐fire (LIF) function is obtained when the current from the TE‐sensor is sufficient to allow the voltage between the collector and the base above the latch‐up voltage Figure 12b‐ii. Once the condition is met, charge carriers flow from the collector to the emitter, triggering a transition to the low‐resistance state. This phenomenon corresponds to the latching effect, in which the transistor “turns on” and permits current to flow from the emitter to the collector. The inclusion of a parasitic capacitor between the collector and ground (Figure 12b‐ii) results in charging and discharging cycles governed by the latch‐up voltage and giving rise to voltage spikes at the output. As pressure increases, the output voltage of the BJT exceeds the latch‐up voltage more frequently, resulting in more rapid transitions between high and low‐resistance states and causing higher frequency spikes in the output, leading to a dynamic output behavior (Figure 12b‐v‐vi). In a subsequent work, Lee et al. [96] showed a hardware implementation of a tactile perception system based on an array of four TE‐sensors serially connected to four biristors for spikes generation and encoding. The parasitic capacitor was removed, and a resistor was connected in series to the emitter. The TE‐sensor area was reduced to the size of a pen tip and that could still trigger the biristor and the encoding process (force range 2–60 N, compatible with keyboard tapping). The recorded spiking signals were used to train a SNN for gesture recognition, achieving an accuracy rate of 92,5 %. Additionally, the system functioned as a self‐aware sensor, activating only in response to external stimuli.

Table 2 summarizes the device structure of both sensor units and neuromorphic devices, as well as pressure or displacement sensitivity and energy consumption for the different triboelectric effect‐driven tactile sensors. Their operational mode and the type of integration of the two units are also indicated.

**TABLE 2:** Neuromorphic Tactile systems based on TE‐sensors. Reported sensitivity is from the TE‐sensor unit if not otherwise stated. Acronyms: SNDP, spikes number dependent plasticity; SDDP, spike duration time‐dependent plasticity; SADP, spike amplitude‐dependent plasticity. The acronym Flex (flexibility) and Stretch (stretchability) are indicated for fully stretchable and/or flexible systems, TE‐sensor and neuromorphic devices are made on or made off stretchable/ flexible components.

TE‐sensor components	Neuromorphic device	Neuromorphic Structure	Integration	Operation mode	Type of synaptic plasticity	Sensitivity (force range)	Mechanical property	Energy / spike	Drain‐source voltage (V_DS_)	Refs.
Cu‐PDMS / Cu	EG‐OFET	Au(S/D)/PVDT‐10/IL/ SiO_2_‐Si	ex situ	Pulse driven	STP, LTP, SNDP	0.192 kPa^−1^ (1‐5 kPa) 0.007 kPa^−1^ (5‐20 kPa)	—	N/A	± 1 V	[76]
FEP / Cu	FG‐FET	Au‐NP, MoS_2_, Si/SiO_2_	ex situ	Displ.driven	STP, LTP, SNDP, SADP, SDDP, SRDP	N/A	—	N/A	0–90 V	[77]
PDMS‐Ag‐NW‐PDMS‐Mxene / skin	EG‐OFET	Au(S/D)/PVDT‐10/IL/ SiO_2_‐Si	ex situ	Pulse driven	STP, LTP, SADP, SDDP, SRDP	0.197 kPa^−1^ (<6 kPa) 0.003 kPa^−1^ (6–30 kPa)	—	N/A	0.5 V	[97]
PET‐Ag / Si‐rubber	EG‐OFET	Au(S/D)/pentacene/gelatin/SiO_2_‐Si	ex situ	Pulse driven	STP, LTP, STDP SNDP	0.98 kPa^−1^ (1‐10 kPa) 0.11 kPa^−1^ (up to 350 kPa)	—	N/A	−1 V	[82]
PDMS‐PMAA / ITO	Memristor	Au/TaO_x_/Ag	direct bias	Pulse driven	STP	0.524 N^−1^ (0.2–2 N) 0.0066 N^−1^ (2–15 N)	Stretchable	N/A	N/A	[93]
Al / PTFE	biristor	Si‐based p‐n junction	direct bias	Pulse driven	STP, SNDP, STDP	N/A	—	0.98 nJ	—	[95]
PDMS / FBP (FBP=1H,1H,2H,2H‐perfluorooctyltrichlorosilane/BaTiO3)	EG‐OFET	Au(S/D)/P3HT/PVDF‐TrFE‐IL/SiO_2_‐Si	full‐bridge rectifier	Pulse driven	STP, LTP	0.38 kPa^−1^ for 3 layers TENG^a^ (0.098–9.8 kPa) 0.01 ‐ 0.02 kPa^−1^ (9.8–98 kPa)	—	N/A	−0.05 V	[86]
Al / PDSM	Si‐transistors	N/A	integrated circuit	Displ. driven	STP, STDP	N/A	—	28.6 mW	5–10 V	[98]
Polyimide / skin	Fe‐OFET	Au(S/D)//pentacene/BaTiO_3_‐PVDF‐TrFE	direct gating	Pulse driven	STP, LTP, SADP, SNDP, SDDP	N/A	Flexible	N/A	−10 V	[87]
Kapton‐Au / ion‐gel	Vertical EG‐OFET	Au(S/D)/Mxenes/PVDT‐10/ion‐gel	direct‐ gating	Displ. driven	STP, SNDP	0.0334 µm^−1^ (0–450 µm) [Integrated sensor 23.75µm^−1^ (75–400 µm)]	Flexible	N/A	−0.5 V	[88]
Al‐PTFE / Al	EG‐FET	Au(S/D)/MoS_2_/IL/SiO_2_‐Si	extended gate	Pulse driven	STP, LTP, STDP	12.3 kPa^−1^ (∼0–40 kPa) 3.7 kPa^−1^ (∼40–120 kPa)	Flexible	11.9 fJ	1 mV	[73]
EGaIn‐PDMS / PDMS‐EGaIn	EG‐OECT	EGaIn(S/D)/PEDOT:PSS/ Na‐hydrogel	extended gate	Pulse driven	STP, LTP, SADP, SNDP, SDDP	0.04 kPa^−1^ (0.24–23.6 kPa)	Flexible, Stretchable	5–6 µJ	−0.1 V	[99]

^a^ 0.23, 0.38, 0.19 kPa^−1^, for 2, 3, 4‐layer TENG, respectively (force range 0.098–9.8 kPa).

### 7.2 Mimicking Complex Biological Functions

**FIGURE 13:**
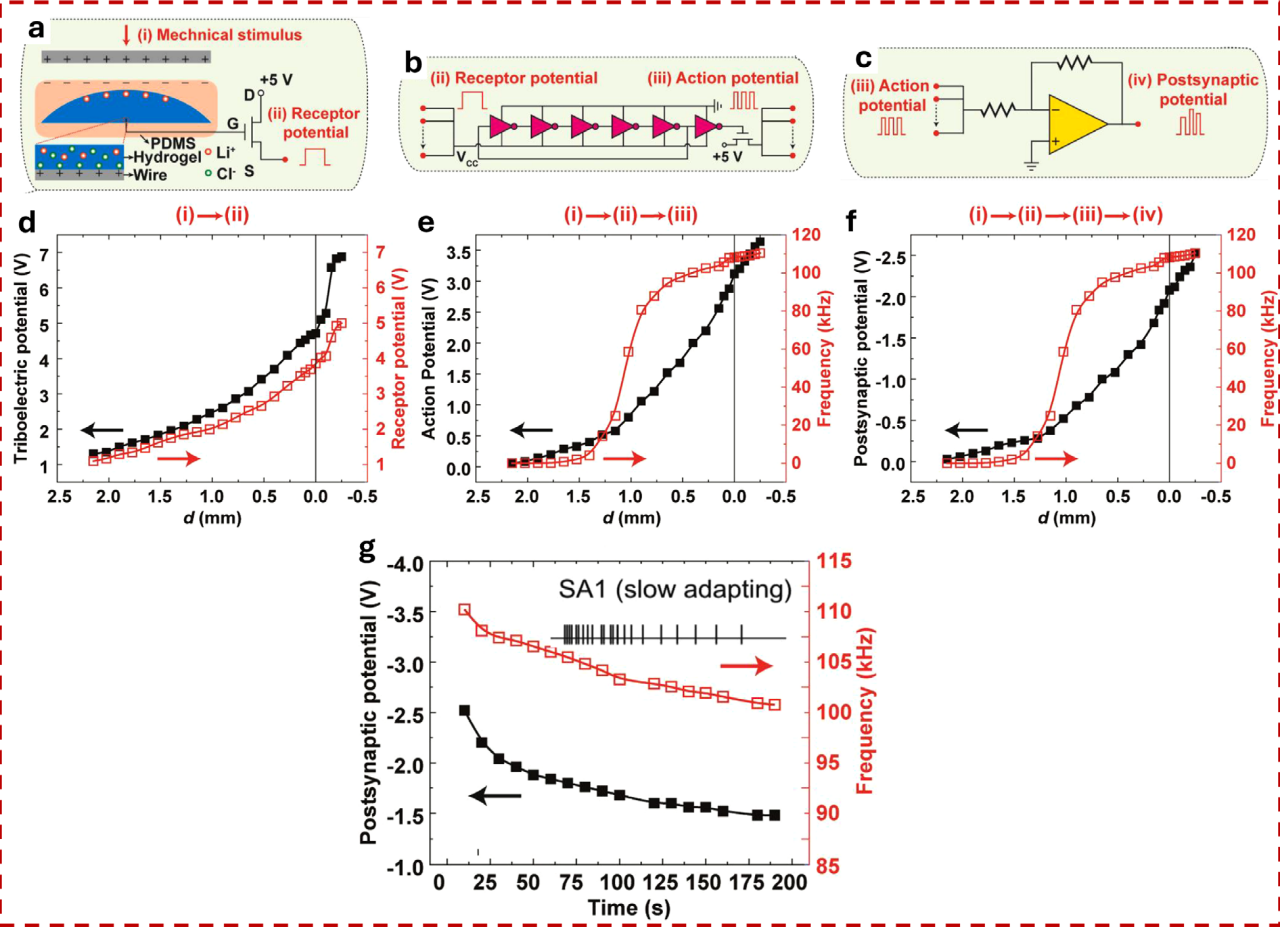
(a–c) Equivalent circuit scheme showing the artificial reproduction of the synaptic mechanoreceptor (a) based on the integration of a TE‐sensor working in the single electrode mode and a transistor, the ring oscillator (b) delivering a train of action potential pulses to an inverting amplifier circuit (c). (d) Tribopotential generated from the TE‐sensor (hydrogel‐based single electrode TENG) and the potential drop recorded as gate voltage of the first layer synapse (receptor potential). The neuromorphic tribotransistor is operated in displacement more, where contact‐and‐press events are recorded at d < 0 mm. (e) Output voltage and frequency of the pulsed action potential generated by the oscillator. (f) Output voltage and frequency of the post‐synaptic potential generated as output of the inverted. (g) Time evolution of magnitude and frequency of the postsynaptic potential during static contact (d= −0.25 mm), demonstrating the pulsed response that emulates the SA‐I slow‐adaptation (inset) [98]. Reproduced with permission [98]. Copyright 2021, Elsevier.

The four types of biological mechanoreceptors described in Section 2 encode and deliver different sensory outputs to the brain, also conveying information about slow adaptation (SA) and fast adaptation (FA) [100]. In humans, SA receptors are based on Meissner and Pacinian corpuscles, while the FA function is accomplished by the Merkel cells and the Ruffini cylinder. Artificial sensors that can mimic the FA function are triboelectric and piezoelectric sensors, due to their fast response to rapid changes in mechanical forces (dynamic sensing). In contrast, the SA function is typically mimicked by capacitive, resistive, and potentiometric sensors, as they can maintain a constant signal during steady mechanical input (static sensing). Hybrid sensors are often designed to cover both functions in artificial systems [101, 102]. Chen et al. [98] developed a hardware‐based biomimetic artificial system capable of reproducing the SA properties of Merkel cells (SA‐I cells). The system generates high‐frequency spiking at the onset of a tactile stimulus, followed by a gradual decrease in spike rate during sustained contact, consistent with the adaptive response observed in biological mechanoreceptors (inset in Figure 13g). The slow adaptive artificial afferent nervous system comprises a tribotransistor (Figure 13a) that emulates the Merkel cell and first synaptic connection, generating post‐synaptic pulses in response to tactile input. These pulses are fed into a ring oscillator circuit (Figure 13b), mimicking first‐order neurons responsible for spike initiation. A subsequent inverting amplifier circuit (Figure 13c) emulates a multi‐synaptic second‐order neuron layer, integrating action potentials from the first‐order neurons. The synaptic connection, as well as the first‐ and second‐order neuron circuits, were implemented on a printed circuit board using standard Si‐electronic components, requiring external power to operate. Despite the complexity of the integrated circuit, the system successfully reproduced the slow‐adaptive behavior of the Merkel cells (Figure 13g). In a follow‐up study, the authors demonstrated advanced coding of dynamic and rapid touch and grasp information by converting the analog output of the TE‐sensor into spike trains. This enabled signal transmission through a tactile SNN, achieving a temporal resolution on the order of a few milliseconds [103].

**FIGURE 14:**
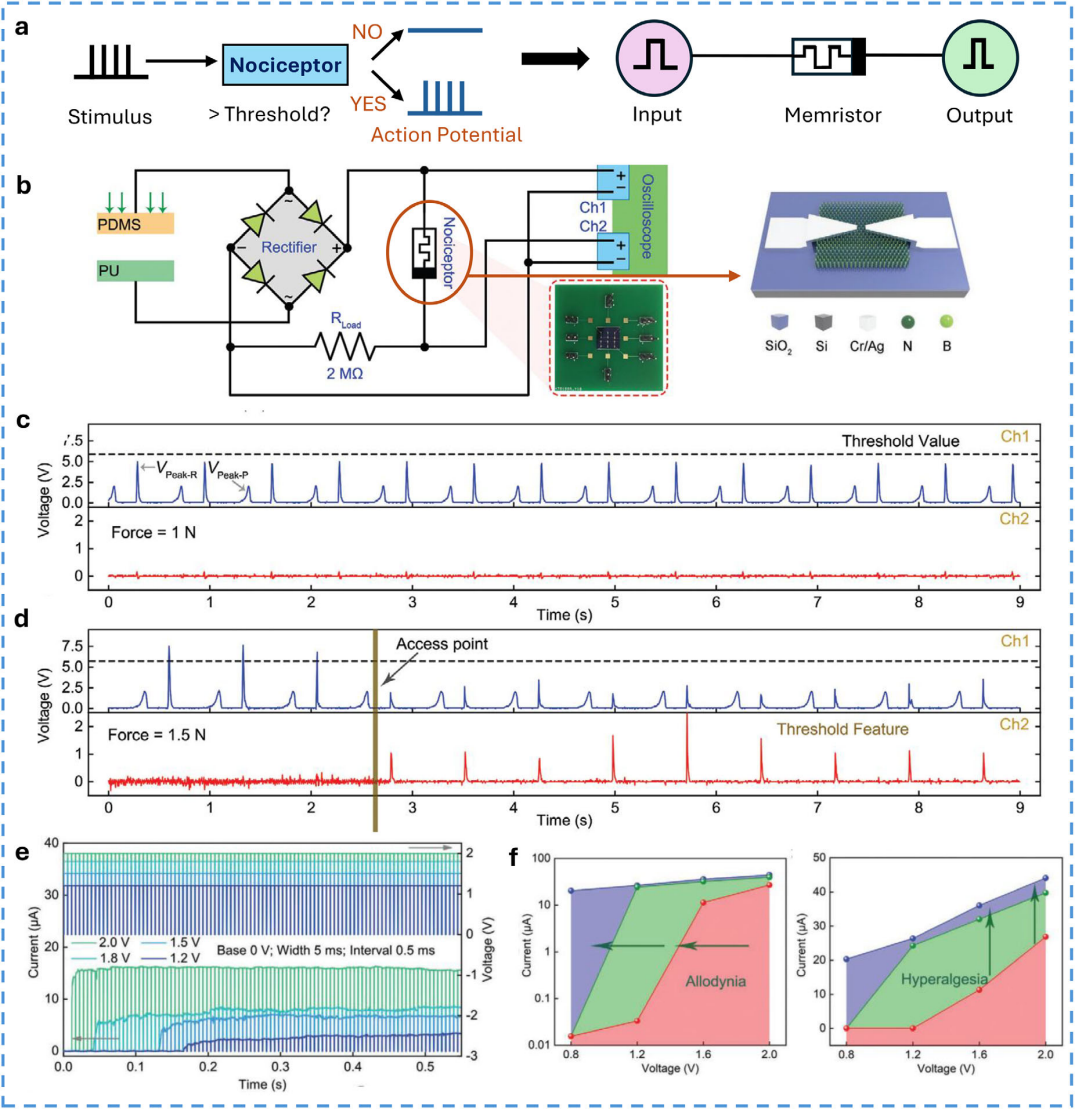
(a) Simplified diagram of the biological nociceptor and its corresponding artificial device based on memristors. (b) Circuit diagram of a self‐power artificial nociceptor system, optical image of memristor nociceptor array, and scheme of the HBN memristor nociceptor. Channel 1 (Ch‐1) of the oscilloscope monitors the voltage output from the TE‐sensor, while the voltage drop on the load resistor (R_load_) is monitored at channel‐2 (Ch2). Ch2 is sensitive to the ON or OFF switching of the memristor nociceptor (pain or no‐pain functions). The voltage output at Ch1 and Ch2 under the force stimulus of (c) 1 N; (d) 1.5 N. (e) Memristor firing threshold dependence on applied external force and the corresponding train of pulses transduced by the TE‐sensors. (f) Characteristic response of the artificial nociceptor showing the emulation of the biological nociceptive functions of allodynia and hyperalgesia [106]. Reproduced with permission [106]. Copyright 2022, Wiley.

The presence of threshold switching followed by a stable storage of data information makes the memristor more prone to be integrated in tactile nociceptors. In biology, nociceptors are sensory receptors that encode noxious stimuli and send neuronal signals that make the brain aware of the possible presence of a threat. In an artificial nociceptor, all information related to non‐noxious tactile events shall be filtered out, therefore they shall not be encoded (Figure 14a). Four signature bio‐nociceptive characteristics should be emulated: i‐ threshold triggering, setting a level above which a warning signal shall be transmitted to the central node; ii‐ no‐adaptation function where continuous triggering of a signal occurs in the presence of a harmful stimuli, with the system not adapting to changing its sensitivity to the external stimuli; iii‐ relaxation upon removal of the noxious stimuli, where the system returns to baseline sensitivity after the harmful stimulus is removed; iv‐ sensitization characterized by a lowering of the threshold in presence of an injury (allodynia) and an intensification of the output (hyperalgesia) according to the strength, duration and repetition of the external stimuli [40]. Threshold switching voltage in diffusive (volatile) memristor and the time‐dependent decay of their stimulated output are typically used to emulate the threshold and relaxation function of a biological nociceptor [104, 105]. The tunable threshold switching voltage induced by electrical stress, such as from continuous or too excessively intense stimuli, can be linked to sensitization functions. The pain perception is emulated when the pulse amplitude is higher than the threshold voltage of the memristor, and the memristor is turned ON (set to low resistance states). The increase in current output corresponds to a harmful stimulus response. Ding et al. [106] fabricated a self‐powered mechano‐nociceptor by connecting a TE‐sensor (2‐electrodes configuration, PDMS and polyurethane (PU) as tribomaterials) to a threshold and planar memristor (Ag/*h*‐BN/Ag MIM structure) through a commercial rectifier bridge to perform the full wave rectification (Figure 14b). A resistor is included in the circuit to measure the voltage drop, which varies according to the switching status of the memristor. The voltage drop is lower when the memristor is switched on. Figure 14c,d shows the correspondence between the input tribopotential pulses with an amplitude either below or above threshold voltage and the corresponding recorded PSC. The volatile memory is triggered for tribopotential pulses delivering > 6 V (pressure > 1 N), corresponding to no‐firing and no‐feeling pain. At higher pressure loads (> 1.5 N, Figure 14d) the tribopotential is above the memristor threshold potential, and the memristor can be fired. This first feature shows how non‐noxious tactile pressure stimuli are filtered out, i.e., they are not recorded in the volatile memory. The TE‐sensor active area, input force, and frequency are parameters that can be adjusted to deliver tribopotential pulses below or above the memristor threshold voltage upon continued pulses, as shown in Figure 14e. This characteristic response emulates biological nociceptive functions such as allodynia and hyperalgesia (Figure 14f).

In recent works, the integration of TE‐sensors with organic artificial nociceptors has also been shown. Xu et al. [107] showed a polymeric array of memristive nociceptors based on a MIM structure (Ag/PVDF‐8/Pt). The high switching endurance (> 10^6^ cycles) allowed to overcome typical limitation of organic memristors suffering high “on currents” and poor switching endurance (∼ 10–10^3^ cycles) due to local joule heating and their low thermal conductivity. A basic TE‐sensor composed of graphite electrodes, paper, and scotch tape was connected ex situ to the array's top and bottom terminals. A noxious stimulus represented by a hard hit applied to the sensors was detected by the turn on of an LED integrated into the memristor circuit. When the hard hit delivers a tribopotential above the memristor threshold, the memristor switches to a high‐current state, corresponding to the detection of pain. The intensity of the pain is indicated by the brightness of the LED.

### 7.3 Multimodal Sensors: Mechano‐Photonic Hybrid Systems

The combination of different external stimuli can be used to trigger different neuromorphic functions [13]. Various systems comprising a TE‐sensor and a neuromorphic tribotransistor have shown that light pulses can be coupled to mechanical inputs, where both stimuli can be self‐powered. State‐of‐the‐art approaches showed that the merge of the TE‐sensor tactile information with optical signal carrying visual information allows higher recognition accuracy and excellent environment‐adaptible perception behavior. For example, a mechano‐photonic artificial memory capable of multibit nonvolatile memory was demonstrated by Zhao et al. [77]. The system comprised a floating gate MoS_2_ transistor, embedding an Al_2_O_3_ tunneling layer and graphene as a charge trapping layer, ex situ connected to a TENG (PTFE/Al as a negative triboelectrode and Al positive triboelectrode connected to the Si/SiO_2_ gate). The system was operated in displacement mode and showed a program/erasing current ratio > 10^7^, with retention of 10^5^ s. The large switching ratio and the combined mechanical/optical regulation enabled to access to multimemory states (11 levels corresponding to 3 bits).

**FIGURE 15:**
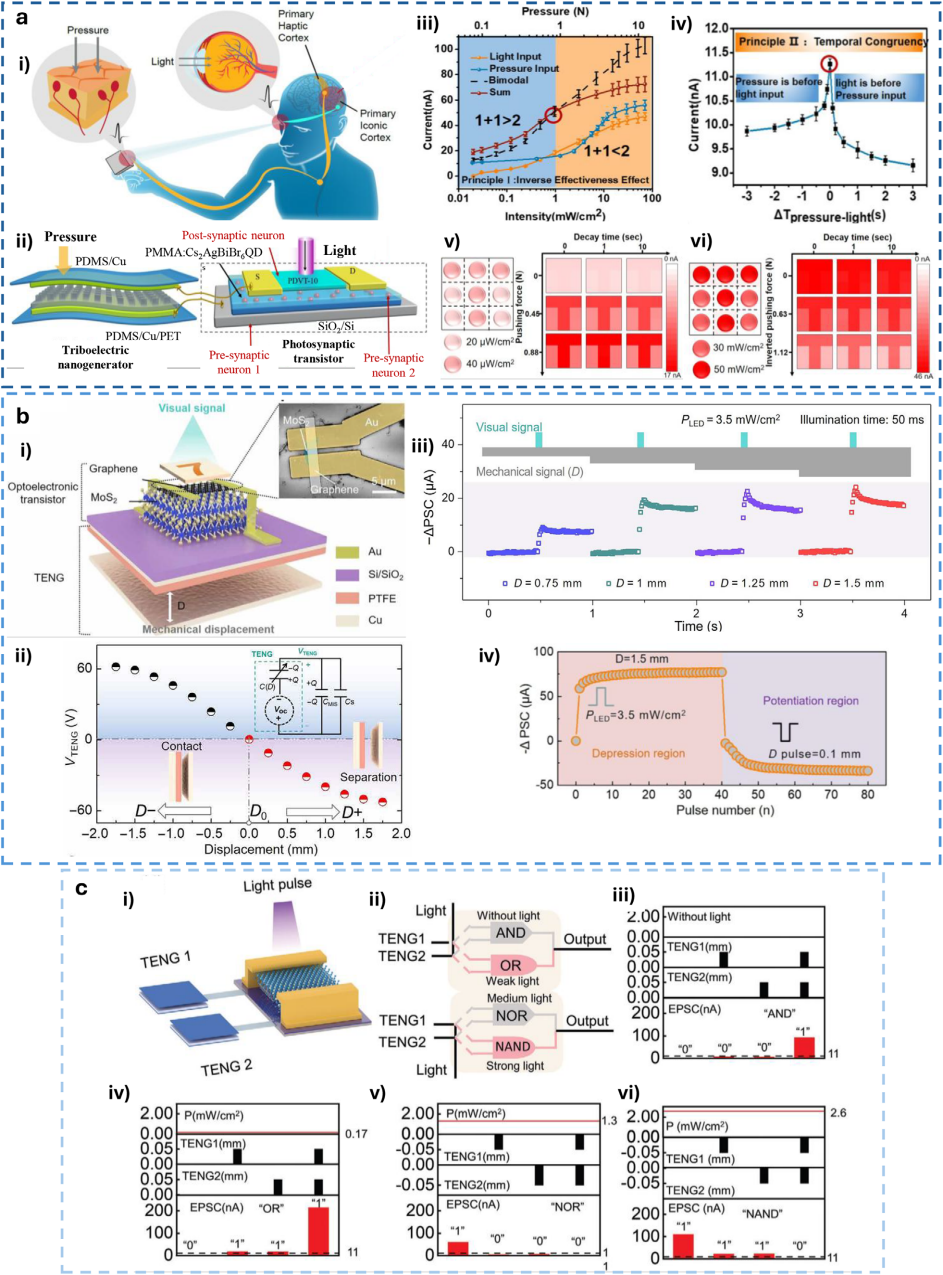
Multimodal artificial tactile system. (a) i‐ Schematic of human haptic and iconic sensing activated in the somatosensory cortex following touch and light inputs. ii‐ Structure of the flexible TENG and floating gate phototransistor integrating light‐sensitive charge trapping layers based on perovskite quantum dots. iii‐ Biomimicry of the “inverse effectiveness” triggered by increasing intensity of light and pressure pulses (the sum EPCS generated from the simultaneous light and pressure inputs shows a transition from higher intensity to lower intensity with respect to the EPSC generated by each type of input). iv‐ Biomimicry of the “temporal congruency” where the EPSC is generated by differently timed (ΔT) light and pressure pulses. Emulation of haptic and iconic perception and improved sensitivity are achieved when pressure inputs are provided in the presence of weak light in a dark background (v), or in the presence of strong light in a bright background (vi) (3x3 phototransistor arrays coupled to 1 TENG, T‐shaped shadow mask) [108]. Reproduced with permission [108]. Copyright 2021, Elsevier. (b) i‐ Scheme of the mechano‐photonic artificial synapse based on graphene/MoS_2_ heterostructure; top image scanning electron microscope (SEM) image of the transistor channel. ii‐ TENG output voltage (*V*_TENG_) versus displacement (*D*) and equivalent circuit for *V*_TENG_ characterization. iii‐ PSC depression (−ΔPSC) recorded under light and displacement inputs (*V*_D_ = 1 V, led power, *P*_LED_ = 3.5 mW cm^−2^, 525 nm, light pulse width 50 ms). iv‐ Depression/potentiation (D/P) curve used for ANN simulation [109]. Reproduced with permission [109]. Copyright 2021, AAAS. (c) i‐ Schemes of the coupled TENGs and synaptic phototransistor, and ii‐ of the logic operations achieved under low and strong light illumination. iii‐ “AND,” iv‐ “OR,” and v‐ “NOR” and vi‐ “NAND” logic executed by illuminating the device with different light powers (365 nm PLED) and operating the two TENGs with different mechanical displacements and timing [110]. Reproduced with permission [110]. Copyright 2024, Wiley.

Very recently, Wu et al. [108] developed an artificial multisensory neural system that combines a quantum dot floating‐gate organic photo‐synaptic transistor with a flexible triboelectric nanogenerator (TENG) based on micro‐patterned PDMS (Figure 15a‐i‐ii). The floating gate phototransistors embedded a lead‐free perovskite (Cs_2_AgBiBr_6_) quantum dot working both as a charge trapping and light‐responsive material. The system exhibited synaptic plasticity in response to both optical and tactile stimuli, enabling artificial perception that combines information from haptic and visual (iconic) sensory modalities. This multisensory capability was demonstrated through the emulation of key features of biological integration systems, such as temporal congruency (Figure 15a‐iv) and the inverse effectiveness effect (Figure 15a‐iii). The integrated optical and tactile stimuli allowed to achieve a recognition accuracy of handwritten digits of ∼ 87% being higher than the one achieved with each type of stimulus. Additionally, the authors demonstrated environment‐adaptable perception behavior showing the improved pattern recognition when tactile stimuli (haptic perception) are combined to light stimuli (iconic perception) in presence of either a weak or strongly bright illumination background (Figure 15a‐v‐vi).

Yu et al. [109]. developed a mechano‐photonic artificial synapse integrating a TENG with a phototransistor based on van der Waals heterostructure composed of a graphene monolayer stacked on a multilayer MoS_2_ (ex situ connection, Figure 15b‐i). The photogenerated charge carrier in the MoS_2_ are transferred to the more conductive graphene layer, which prevents charge recombination and favors photoconductivity. The TENG (Cu as positive triboelectrode and Cu/PTFE as negative triboelectrode) was operated in displacement mode (Figure 15b‐ii), and the generated tribopotential was shown to regulate the charge migration through the MoS_2_/graphene junction. Mechanical displacement influences the initial PSC, while combining mechanical and optical stimuli enhances synaptic plasticity (Figure 15b‐iii), including PPF. Simulation based on artificial neural networks (simulation input data from experimental potentiation/depression data, Figure 15b‐iv), demonstrates that mechanical plasticity improves image recognition accuracy (92%), indicating strong potential for bimodal synaptic plasticity in neuromorphic computing.

Gong et al. [110]. investigated the logic properties of tribopotential and light‐modulated 2D‐Fe‐FETs based on MoS_2_/ferroelectric‐(CuInP_2_S_6_) heterostructures. The device executes “AND” and “OR” operations using two distinct light pulses (365 and 465 nm). Beyond acting as inputs, light pulses also served as modulation signals that control output current for additional logic functions. By integrating two TENGs inputs with a 365 nm light modulation signal, the device demonstrates “AND,” “OR,” “NOR,” and “NAND” logic (Figure 15c). Under dark or low‐light conditions, logic outputs are determined based on whether combined tribopotential displacements exceed or fall below a current threshold (11 nA). Positive displacements enable “AND”/“OR” operations (Figure 15c‐iii‐iv), whereas negative displacements combined with varying light intensities allow for “NOR” and “NAND” logic operations (Figure 15c‐v‐vi). This hybrid triboelectric‐optical approach supports multiple Boolean logic gates within a single neuromorphic device, enhancing the flexibility and performance of artificial neural systems for intelligent applications.

Tactile thermal dual‐mode perception was demonstrated by Huang et al. [111], combining a commercial TEG and a PDMS‐PTFE TE‐sensor, both delivering input to the gate of an oxide neuromorphic transistor (ITO semiconductor, chitosan‐based electrolyte). The authors showed that synaptic plasticity is controlled by the temperature and pressure‐dependent gate voltage.

## 8 Open Challenges and Future Perspectives

Artificial synaptic devices regulated by TE‐sensors have demonstrated strong potential for simulating human sensory functions such as touch, hearing, and taste, while also serving as self‐powered energy sources, significantly reducing power consumption. Many TENG‐based devices already operate within the biological energy range (1–100 fJ per event), making them viable for low‐power neuromorphic applications. These devices can be integrated with synaptic and neuronal circuits and have shown effective data storage and plasticity simulation. These achievements have a large potential impact on applications for the next generation of human‐machine interfaces, robotics, prosthetics, and wearables. However, TENGs still require improvements both from the material perspective, device architecture design, and system integration.

### 8.1 Stability, Sensitivity, and Environmental Robustness

#### 8.1.1 Improving Stability

A key factor limiting long‐term stability is the screening of triboelectric surface charges by adsorbed water layers. Under humid conditions, water molecules absorb on the tribomaterial where their dipoles reorient in response to the local electrostatic field generated by the triboelectric charges. This dipolar rearrangement partially screens the surface potential, while protonic or ionic transport within the water layer can further accelerate charge dissipation. Encapsulation can be another effective approach for two‐electrode TENGs, but design considerations like spacers and elastic scaffolds are also necessary. Several non‐hermetic strategies, such as the use of hydrophobic or fluorinated polymers, nano‐/micro‐textured surfaces to reduce wet contact area or passivation layers, can effectively suppress humidity‐induced signal decay while preserving mechanical flexibility.

#### 8.1.2 Improving Sensitivity and Linearity Range

Although TE‐sensors exhibit high sensitivity in the low‐force range (up to a few tens of Newtons), their sensitivity significantly decreases at higher pressures, resulting in a reduced dynamic range as high sensitivity and good linearity are primarily confined to the lower force regime. So far, strategies have explored the use of multilayered and porous tribomaterials have been used to enlarge the dynamic range and to extend force sensitivity to higher force loads. Future improvements are expected through material innovations and device structural design, including the use of emerging materials like graphene, MoS_2_, WSe_2_, and biodegradable biomaterials (e.g., chitosan, RNA), which offer advantages in performance and biocompatibility.

### 8.2 Integration and Miniaturization

#### 8.2.1 Improving Device Architecture to Facilitate Integration

Though the two‐electrode configuration ensures higher sensitivity and stability, to enable easier integration into real‐world applications, TE‐sensors with a single electrode configuration are more desirable. Achieving full integration and minimizing the architectural complexity of TE neuromorphic sensors requires the development of monolithic integration strategies, in which the TE‐sensing and neuromorphic components are combined into a single platform, thereby removing the need for external interconnects and auxiliary circuits like rectifiers. This approach will lead to a reduction of hardware components, reaching high speed and greatly lowering energy dissipation for both device operation and data transmission.

#### 8.2.2 Getting Closer to Realistic Tactile Operation Conditions

Though devices operated in displacement mode typically achieve strong synaptic behaviors under very small displacements (tens of µm up to a few mm), the slow speed and small displacement is quite far from reproducing a realistic human touch. This makes event‐driven neuromorphic TE‐sensors more prone to emulate tactile sensation, and efforts to improve their integration into neuromorphic devices and artificial networks should be intensified.

#### 8.2.3 Ensure High Sensitivity and Energy Harvesting Upon Taxel Downscaling

Another important matter is the need to maximize the mechanical energy conversion efficiency upon downscaling of the sensors active area. An inherent limitation of triboelectric sensors is the need to maintain sufficient surface contact area for effective tactile response. To date, taxel areas as small as a few mm^2^ have demonstrated adequate performance, enabling their integration into practical applications. Along with increasing taxels resolution, issues originated by taxels cross talks and proximity effects in sensor arrays shall also be considered during design and testing.

### 8.3 Performance Variability and Lack of Standardization

The emerging field of TE neuromorphic sensors also needs to face several limitations of TENGs field as related to data inaccuracy, particularly concerning performance dependence on type of materials and applied force range, as well as the lack of a standardization protocols for materials, mechanoelectrical testing systems and electrical performance, which can lead to inconsistent results and difficulty in comparing research findings across different studies. While TENG output generally increases with applied force, the relationship is not linear over large force ranges. This can lead to discrepancies in interpreting results, especially when comparing data from different studies with varying force application protocols. To overcome these issues, it will be essential to (i) establish community‐wide standard testing procedures for force profiles, contact areas, and environmental conditions; (ii) adopt unified figures of merit such as open‐circuit voltage, transferred charge, and energy per event; and (iii) correlate device‐level electrical outputs with system‐level performance metrics.

### 8.4 Bio‐Inspired Designs and Algorithm‐Hardware Co‐Design

#### 8.4.1 Improve Biomimicry

Hybrid systems that can seamlessly integrate both SA and FA functions while minimizing energy costs are key to advancing the next step in human integration. In addition, adaptive and stretchable structures that mimic biological systems and respond to environmental stimuli can further improve TE‐sensor functionality. Advances in surface microstructures and bio‐inspired designs (e.g., conformal and responsive to curved surfaces) also show promise in enhancing dynamic sensitivity and energy harvesting capabilities. Actionable strategies include the integration of kirigami/stretchable meshes and self‐healing polymers that maintain contact quality even under large deformations.

#### 8.4.2 Artificial Network Co‐Design

Beyond hardware, the integration of machine learning and neural network algorithms with TENG‐based synaptic devices is critical. Current models are not optimized for the unique interface of self‐powered TENG systems, necessitating customized algorithm development. Such integration can improve autonomous learning, reduce training time and cost, and enhance real‐time data processing and decision‐making in applications like IoT, smart cities, healthcare, and environmental monitoring. Steps toward this goal include: (i) developing neuromorphic algorithms compatible with spike‐driven, event‐based TE outputs; (ii) leveraging in‐sensor learning rules matched to the dynamics of triboelectric synaptic devices; and (iii) designing hybrid hardware‐software frameworks capable of optimizing sensor characteristics and inference performance.

### 8.5 System‐Level Integration and Energy Efficiency

Moreover, system‐level integration is essential. While much research has focused on individual devices, the realization of a fully functional artificial neural network requires coordinated hardware‐level integration of TENGs and neuromorphic elements. This includes designing scalable arrays and ensuring TENGs can effectively power and modulate multiple interconnected synaptic units.

Next‐generation neuromorphic systems shall comply with specific performance metrics aligned with new technologies requirements including (1) non‐volatile memory, essential to reliably store weight values; (2) high integration to enable scalability of devices arrays; (3) high operating speed to support fast inference (read) and training (write) operations in ANNs; and (4) high energy‐efficiency to minimize power consumption during neural computations.

In memristors, a small programming and erasing current ratio and the short retention time obstruct the further development of multilevel data storage. Low programming/erasing current ratio would not provide distinguishable current states for multilevel data storage (small memory window). Uncontrolled stored charges into the floating gate represent a limitation to reach practical applications. Required characteristics for a tribotransistor: efficient electrostatic(gating) control, high mobility, and scaled/large operating/switching voltage. To reach full energy autonomous neuromorphic systems energy consumption of the neuromorphic unit shall also be targeted. Potential solutions include: (i) engineering stable charge‐trapping layers with controlled defect densities; (ii) adopting ferroelectric or electrolyte‐gated architectures to achieve wider memory windows at low voltages; (iii) minimizing leakage through optimized dielectric stacks; and (iv) implementing energy‐minimization strategies such as event‐driven programming or local energy harvesting buffering.

### 8.6 Multimodal Sensing and Biomimetic Functional Integration

Current artificial sensors often lack the efficiency, integration density, and multifunctionality when compared to their biological counterparts. Future developments should focus on advancing multimodal sensing capabilities by emulating both the sensory and the memory functions of the full range of somatosensory mechanoreceptors and afferent nerves. Targeted goals include achieving high sensitivity, real‐time perception, and seamless integration of multiple modalities. Expanding the functionality of TE‐sensors beyond pressure detection, toward multimodal mechanical sensing, is essential for improving the realism and versatility of artificial touch. Beyond mechanical sensing, the vision is to develop fully integrated platforms in which multiple sensors operate synergistically to sense and compute, ultimately enabling truly biomimetic systems. Actionable approaches include: (i) hybrid integration of mechano‐photonic, mechano‐thermal, and mechano‐chemical sensing elements; (ii) exploiting multifunctional materials capable of encoding diverse stimuli into distinct triboelectric signatures; and (iii) designing hierarchical architectures that fuse multimodal signals at the device level before neuromorphic processing.

In conclusion, despite existing technical challenges, TENG‐regulated neuromorphic devices represent a highly promising route for neuromorphic electronics, combining fast response, high integration potential, fine control, and low energy operation. Continued interdisciplinary progress in materials science, device engineering, and algorithm development is expected to unlock transformative advances across AI, biomedicine, robotics, and beyond.

## Funding

The authors acknowledge the financial support of Ministero dello Sviluppo Economico (MISE) now Ministero delle Imprese e del Made in Italy (MIMI), Fondo per lo sviluppo di tecnologie e applicazioni di intelligenza artificiale, blockchain e internet of things, project FTE0000034, SMARTCAP, C.U.P. B87H21012300008.

## Conflicts of Interest

The authors declare no conflicts of interest.

## Data Availability

The authors have nothing to report.
